# Solving the Conundrum of the Influence of Irradiation Power on Photothermal CO_2_ Hydrogenation

**DOI:** 10.1021/acscatal.5c00247

**Published:** 2025-02-19

**Authors:** Horatiu Szalad, Yong Peng, Jonas Werner Gosch, Andrea Baldi, Sven H. C. Askes, Josep Albero, Hermenegildo García

**Affiliations:** † Instituto Universitario de Tecnología Química (CSIC-UPV), 16774 Universitat Politècnica de Valènica, Avda. De los Naranjos s/n, 46022 Valencia, Spain; ‡ Department of Physics and Astronomy, 1190 Vrije Universiteit Amsterdam, De Boelelaan 1081, 1081 HV Amsterdam, Netherlands

**Keywords:** photothermal catalysis, CO_2_ conversion, plasmonic nanoparticles, finite element modeling, nanoscale temperature

## Abstract

Solar photocatalysis appears as a viable approach for the production of value-added chemicals from CO_2_. However, up to now, there is no information on the influence of the light intensity on the product distribution of CO_2_ hydrogenation and the modeling of the actual local temperature at the catalytic sites for typical nanoparticulate photocatalysts. Herein, it is shown that for a photothermal catalyst containing a high density of homogeneously distributed Ru nanoparticles, the collective heating prevails, resulting in a homogeneous temperature distribution in the material that should be relatively close to that of the support and that can be measured macroscopically. Moreover, light intensity has a clear influence on product distribution due to the differences in the local temperature, and therefore, attention should be paid to stable operating conditions, temperature, and CO_2_ conversion that can result in remarkable differences in product selectivity for the same catalyst as a function of light intensity.

## Graphical abstract

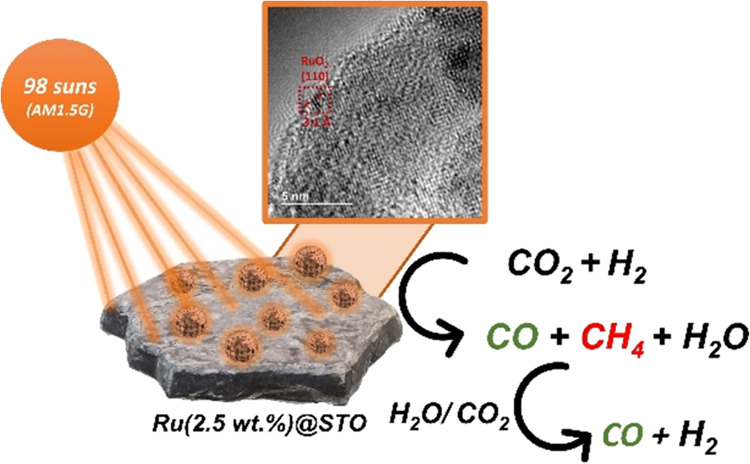

## 1 Introduction

In the context of achieving the targets on atmospheric CO_2_ emissions set by International Climate Summits to limit the average Earth temperature and to minimize climate change,^1^ one of the possibilities consists of CO_2_ capture and utilization (CCU).^2^ In this strategy, CO_2_ is converted into chemicals and fuels, becoming, in that way, a feedstock for subsequent industrial processes. Among the possible reactions, CO_2_ hydrogenation can lead to a wide range of products, from CO^3–5^ and methane^6–10^ to methanol,^11–14^ C_2+_,^15–17^ and aromatics.^18–20^ While catalytic CO_2_ hydrogenation has been known since the early 20th century,^21^ light-assisted CO_2_ hydrogenation has more recently appeared as a viable alternative to pure thermal catalytic reactions.^22^

Regarding photocatalytic CO_2_ hydrogenation, various materials and particularly transition metal nanoparticles (NPs) supported on metal oxides have shown a high efficiency, mainly forming either methane or CO.^23,24^

Photothermal catalysts, consisting mainly of metallic species embedded into adequate supports, have attracted great interest as a viable approach for the valorization of CO_2_, considering the close similarities between vastly investigated heterogeneous systems under dark thermal conditions and these materials whose activity arises from the thermalization of incident photons, therefore converting light energy into chemical energy at the adsorption point. Given that these light-assisted processes are performed and investigated under different light intensities, or even assisted through externally applied heat, a great deal of effort is required for the standardization of testing conditions for such catalysts, the main parameter that tends to vary being the local temperature at the catalyst interface.

Thus, in some cases, the system is heated at temperatures of 350 °C or below, while the photocatalyst is being irradiated with simulated sunlight at standard one sun intensity.^25,26^ In other contrasting reports, concentrated light, frequently equivalent to the power of 20 to 50 suns, is used, while the photocatalyst is not heated by any additional means other than irradiation.^27,28^ These variations in employed reaction conditions may lead to variation in the product distribution for very similar or even the same photocatalytic materials.

One of the most studied photocatalytic systems is based on Ru metal NPs supported on various materials. Using Ru photocatalysts, data from the literature show contrasting preferential product selectivity, leading in some cases to the selective formation of CH_4_ when irradiated at low light intensities and in others to CO when irradiation is performed at high light intensity. Table S1 in the Supporting Information provides a summary of the reported product distribution for similar supported Ru photocatalysts depending on the irradiation conditions. In both cases, either illuminating with one sun intensity and external electrical heating or using highly concentrated light without additional external heating, a photothermal mechanism has been claimed. In this mechanism, photon energy is converted into heat at the nanometric scale by the metal particles.^29^ The uniqueness of the photothermal heating mechanism is that using photons, the heat is generated at the metal nanoparticle where the reaction takes place without the need to heat the reactor walls and the whole system. In these photothermal systems, it could be possible that the local temperature on the nanoscale on the metal surface could be much higher than the macroscopic temperature measured by conventional thermocouples. Therefore, it would be important to determine the reasons why the light intensity on the CO_2_ hydrogenation influences the product distribution and if the same photocatalyst changes selectivity as a function of light intensity in the 1 to 50 suns power range.^30^ It is also necessary to estimate the local temperatures at the NPs acting as the catalytic center compared to the bulk macroscopic temperature measured with thermocouples. This can be done by using microkinetic simulation models based on Monte Carlo theoretical models and “continuous space models” that have proved to be valid in determining temperature distribution in several other systems and that can also be applied to powdered photocatalysts containing nanoparticulate metal species distributed in a 3D space.^31^ According to these models, depending on the experimental illumination conditions, nanoparticle density, and their absorption cross section, it could be possible to induce either substantial nanoscale temperature gradients or not.^32^ Still, in the latter case micro- to milliscale temperature gradients are not excluded, for instance, due to high light scatter or light source beam shapes. Thus, the potential presence of both nano- and macroscale temperature gradients, and their influence on photothermal reactions, calls for an integrated modeling approach that considers all of these aspects. As far as we know the influence of light intensity on the product distribution and on the local temperature in the photothermal CO_2_ hydrogenation has not been studied before. Thus, in the literature, it is assumed that light intensity varies the product rate, but not the product distribution.^22^ Therefore, it seems convenient to rationalize the different product distributions reported for similar Ru photocatalysts and for advancing on a better control of the efficiency and selectivity of photothermal reactions.

Herein it will be shown that for a powder photothermal catalyst having a high density of homogeneously distributed plasmonic NPs, collective heating prevails, resulting in a homogeneous temperature in the material that should be relatively close to that of the support and that should be measurable macroscopically. In those systems, light intensity may change product distribution due to differences in the temperature at the metal NPs and therefore, photothermal catalysis follows an identical pattern as heterogeneous thermal catalysis, resulting in remarkable differences in product selectivity for the same catalyst.

## 2 Methods and Materials

### 2.1 Characterization

*Powder X-ray diffraction* (PXRD): X-ray diffraction patterns were acquired through a Shimadzu XRD-7000 diffractometer employing a Cu kα irradiation source (λ = 1.5418 Å) operational at 40 kW and 40 mA. All data acquisition was done with a scanning speed of 10°/min in the 2–90 range (2θ°). *X-ray fluorescence* (XRF): The ruthenium content in Ru@STO was determined by employing X-ray fluorescence via an XRF Philips MiniPal 25 fm instrument. *Transmission electron microscopy* (TEM): TEM images were recorded via a JEOL JEM 2100F microscope operating under an accelerating voltage of 200 kV. The samples were uniformly dispersed in an ethanol solution and subsequently drop-cast on Ni TEM grids, which were allowed to dry at room temperature. The supported samples were thereafter investigated via a TEM microscope. *X-ray photoelectron spectroscopy* (XPS): XPS data was acquired via SPECS equipment with a Phoibos 150 MCD-9 detector. The X-ray source was either Al or Mg (monochromatic), and it was operated at a power of 200 W. Before the acquisition of sample spectra, the measurement setup antechamber was evacuated at 10^–9^ mBa. The work function of the measurement device was calibrated via Ag, Au, and Cu premade standards, and the value obtained was 4.2440 eV. The intensity ratios of components observed in analyzed samples were obtained via Shirley-type background subtraction and correction by the transition function of the spectrometer. *Transient absorption spectroscopy* (TAS): Transient absorption spectra were recorded using the fourth harmonic of a Q switched Nd:YAG laser (Quantel Brilliant, 266 nm, 15 mJ/pulse, 7 ns fwhm) coupled to mLFP-122 Luzchem miniaturized detection equipment, the device being equipped with a 300 W Xe lamp, a monochromator (125 nm), a Tektronix TDS-2001C digitizer, compact photomultiplier and power supply, cell holder and fiber-optic connectors, computer interfaces, and a software package developed in the LabVIEW environment from National Instruments. The laser pump is able to generate a 5 V trigger pulse, which can be further adjusted in terms of frequency and delay. The laser pulse is probed by a fiber that synchronizes the photomultiplier detection system with the digitizer operating in the pretrigger mode.

### 2.2 Catalyst Preparation

The herein reported Ru/STO was prepared via a modified method previously reported.^1,2^ Therefore, 1 g of a commercial SrTiO_3_ support (strontium titanate nanopowder, 30 nm particle size) was dispersed in 20 mL of deionized water by ultrasonication (30 min). To this fine dispersion, a specific amount of Ru^3+^ precursor (RuCl_3_·*x*H_2_O, 98%) corresponding to a final 2.5 wt % Ru loading was added. This mixture was then left in a sonication bath for around 30 min to ensure uniform Ru incorporation and was thereafter slowly evaporated under vigorous stirring (60 °C). The recovered solid was left to further dry in a 100 °C electrical oven overnight. The material was then calcined at 250 °C (5 °C min^–1^) for 2 h, followed by a reduction procedure under pure H_2_ (99.99% purity) at 350 °C (5 °C min^–1^) for 2 h. Under the same synthetic procedure, a Ru/Al_2_O_3_ analogue was prepared, employing a commercial Al_2_O_3_ support (<50 nm particle size).

### 2.3 Photothermal CO_2_ Hydrogenation Tests (Flow)

In a general procedure, 100 mg of catalyst was carefully placed in the center of an aluminum body reactor equipped with a quartz windowed lid. The system was thereafter connected to a continuous flow of CO_2_ (13 mL·min^–1^) and H_2_ (13 mL·min^–1^) without a build of any overpressure in the system (operation under atmospheric pressure) and left to evacuate the rector for 30 min. Thereafter, AM1.5G filtered simulated light generated via a PLS-SXE300E (PerfectLight) 300 W Xe lamp with variable current density was employed as both an irradiation and heat source. The system was left to reach equilibrium under continuous irradiation and gas feed for 1 h, after which product evolution was monitored.

### 2.4 Photothermal CO_2_ Hydrogenation Tests (Batch)

The same reactor system previously described was carefully loaded with the photocatalyst and evacuated with pure H_2_ (99.99% purity) for 30 min, after which 0.6 ba of both H_2_ and CO_2_ (1:1 v/v) were loaded in the reactor. After this, the system was subjected to irradiation from AM1.5G filtered simulated light generated via a PLS-SXE300E 300 W Xe lamp (9.86 W·cm^–2^). Product evolution was monitored at specific reaction times.

### 2.5 Photothermal CH_4_ Steam Reforming Tests (Batch)

In a similar manner as for CO_2_ hydrogenation, the reactor was loaded with 100 mg of Ru@STO with an additional 100 μL of deionized water and evacuated with an Ar flow for 30 min. The reactor was pressurized with 0.9 bar of Ar and an additional 0.1 bar of CH_4_ gas (10 vol %). After this, the system was subjected to irradiation from an AM1.5G filtered simulated light generated via a PLS-SXE300E 300 W Xe lamp (9.86 W·cm^–2^). Product evolution was monitored at specific reaction times.

### 2.6 Photothermal CH_4_ Dry Reforming Tests (Batch)

Similar to the other batch regime tests, 100 mg of photocatalyst was loaded into the reactor described herein, after which the system was evacuated with an argon flow. The reactor was pressurized with 0.8 bar of Ar together with 0.1 bar of CH_4_ and CO_2_, respectively. After this, the system was subjected to irradiation from an AM1.5G filtered simulated light generated via a PLS-SXE300E 300 W Xe lamp (9.86 W·cm^–2^). Product evolution was monitored at specific reaction times.

### 2.7 Thermocatalytic CO_2_ Hydrogenation Tests (Dark)

In a similar manner, 100 mg of Ru@STO were placed in a tube quartz reactor surrounded by a heating mantle and connected with a mantled preheater at the inlet. The system was connected to a continuous flow of CO_2_ (13 mL·min^–1^) and H_2_ (13 mL·min^–1^) without a buildup of any overpressure and left to evacuate the reactor for 30 min. The reactor was thereafter heated to desired temperatures, and before product analysis, the system was left to reach equilibrium under gas feed and heat for 1 h.

Products quantification: The evolved gases were analyzed using a gas chromatograph (Agilent 7890A) equipped with a Carboxene 1010 column analyzing CO_2_, CO, and up to C_3_ hydrocarbons. Quantification of the percentage of each gas was based on prior calibration of the system injecting mixtures with known percentage of gases.

## 3 Results and Discussion

### 3.1 Photocatalyst Synthesis and Characterization

In the present study, a composite composed of Ru NPs supported on SrTiO_3_ (Ru/STO) was employed as a photocatalyst. Previous literature reports have shown that this supported Ru photocatalyst is highly efficient for CO_2_ hydrogenation to methane under one sun power irradiation (0.1 W·cm^–2^) with simulated sunlight and at temperatures about 150 °C or higher.^10^ Continuing with this material, the target is to determine the influence of light intensity and temperature on the performance of Ru/STO as a photocatalyst, particularly the influence on product selectivity.

Herein, we have prepared Ru/STO following a previously reported procedure,^10^ aiming for a Ru loading of 2.5 wt %, which was thereafter confirmed via quantitative X-ray fluorescence spectroscopy (XRF). The process consists of impregnation of a commercially available STO powder with an aqueous solution of RuCl_3_. The suspension was stirred until complete water evaporation at 50 °C, and the resulting powder was calcined at 350 °C before treatment with pure H_2_ atmosphere at 450 °C to perform chemical reduction. The synthesis process is illustrated in Scheme 1.

**Scheme 1:**
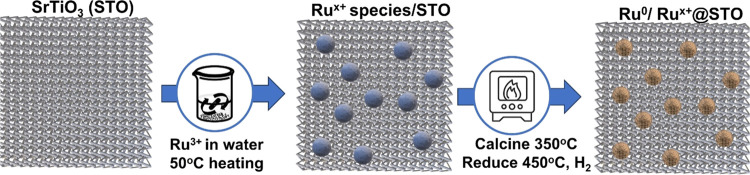
Illustration of the Synthesis of the Herein Studied Ru/STO Photocatalyst

To assess the structural nature of the Ru/STO photocatalyst prepared Ru/STO photocatalyst, powder X-ray diffraction (PXRD) patterns were acquired (Figure 1a). Recorded data did not show any specific diffraction line characteristic to Ru^0^/Ru^x+^ species, the analyzed material showing only diffraction lines attributed to crystalline facets of SrTiO_3_ (PDF #47–0226). Hence, all Ru^0^/Ru^*x*+^ species attributed to the present 2.5 wt % metal loading should be well dispersed with a mean particle size smaller than 2 nm and, therefore, undetectable by conventional PXRD. This assessment agrees with the recorded transmission electron microscopy (TEM) images of Ru/STO presented also in Figure 1b. Selected TEM images of this solid show very fine Ru NPs of sizes around 1 nm diameter (histogram illustrated in Figure S1) homogeneously dispersed on much larger SrTiO_3_ particles of about 20–40 nm. High-resolution images of the fresh photocatalyst show NPs exhibiting well-defined facets with a fringe distance of 3.1 Å that corresponds to (110) facets of RuO_2_ (PDF #43–1027). We attribute the presence of RuO_2_ on top of metal Ru NPs to spontaneous surface passivation upon ambient storage typically occurring in metal nanoparticles of such low sizes. X-ray photoemission spectroscopy (XPS) measurements were performed to further confirm the oxidation states of Ru. Figure S2 presents the XPS spectrum acquired from Ru/STO. As can be seen, the deconvolution from Ru 3p + Ti 2p spectra confirms the presence of metallic Ru^0′^ species in the NP surface with a binding energy of 460.72 eV, together with RuO_2_ (462.61 eV).

**Figure 1:**
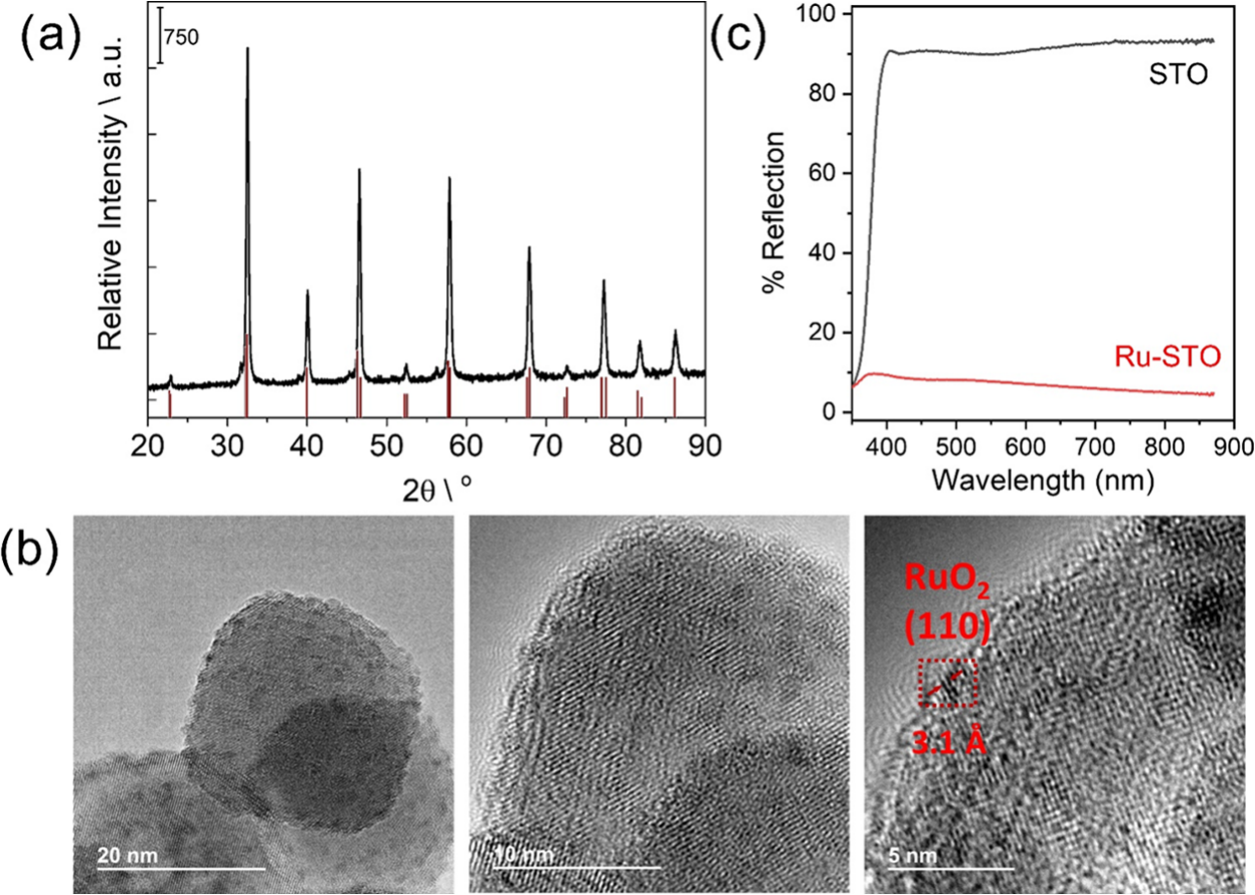
(a) PXRD pattern recorded for Ru/STO and the simulated lines specific for SrTiO_3_ (PDF #47–0226). (b) Selected TEM images of Ru/STO and *d*-spacing associated with RuO_2_ (PDF #43–1027). (c) Diffuse reflectance spectra (after correction) for STO (black) and Ru-STO (red).

Additionally, peaks at 458.29 and 464.13 eV have been attributed to Ti^4+^. The influence of Ru NPs in the considered Ru/STO composite can be clearly observed in the recorded diffuse reflectance UV–vis–NIR spectroscopy data, which is also illustrated in Figure 1c. Diffuse reflectance spectrum obtained for SrTiO_3_ corresponds to strong interaction with UV-light of contributions up to 380 nm. Interestingly, when Ru NPs are embedded into the matrix, a very broad contribution extending into the NIR region is observed in the recorded reflectance spectrum, which is in good correlation with the change in the visual appearance of the solid, from the white color associated with pure phase SrTiO_3_, to dark gray in the case of Ru/SrTiO_3_. We attribute this broad signal to the combined light absorption arising from both Ru and RuO_2_ species present in the sample under study. This feature is important in understanding photoresponse of the employed Ru/STO photocatalyst.

### 3.2 Photocatalytic Results

Light-assisted catalytic measurements were carried out under a continuous flow using a CO_2_/H_2_ ratio of 1:1, while a thin bed of the photocatalyst (1 mm thickness, uncompressed powder) is illuminated with concentrated simulated sunlight (AM 1.5G optical filter, 0.25 cm^2^ irradiation spot). The results presented herein were obtained for various light intensities with powers ranging from 3.97 to 9.86 W·cm^–2^ (Figure 2a).

**Figure 2:**
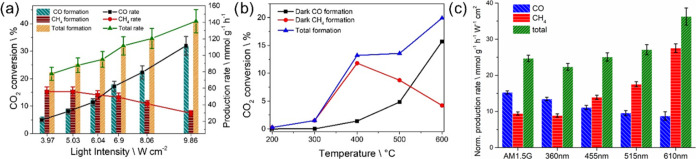
(a) Catalytic output of CO_2_ hydrogenation using Ru/STO as photocatalyst upon increasing light irradiation power, the corresponding CO evolution is indicated with cyan bars, wine red bars correspond to CH_4_ production and orange bars to the total CO_2_ conversion; the corresponding production rates are illustrated with black squares (CO), red circles (CH_4_) and green triangles (total conversion). (b) Catalytic tests carried out in the dark by external heating, CO production is indicated with the black squares, CH_4_ production is illustrated with red circles, and total CO_2_ conversion with blue triangle. (c) Plots of CO and CH_4_ production rates normalized by light intensity for the light-assisted CO_2_ hydrogenation carried out under different light contributions measured using long-pass cutoff filters that stops radiation of wavelength shorter than the nominal value, corresponding production rates are indicated with cyan blue (CO), wine red (CH_4_), and green (total CO_2_ conversion) bars. Reaction conditions: CO_2_/H_2_ ratio 1:1, gas flow 26 mL min^–1^, Ru/STO amount 100 mg, and light intensity as indicated in Table S3 for each filter. Rates of evolved gases were normalized by the light power for each spectral range (W·cm^–2^).

This high-power light is achieved using a 300 W Xe lamp equipped with concentration lenses. The lenses are a fixed component in the lamp, and therefore, the beam spot area is constant, independently of the light intensity output, which depends exclusively on the current density applied to the Xe lamp. Depending on the current intensity range in which the Xe lamp can be operated, the light intensity varies from 3.97 to 9.86 W·cm^–2^ at low and high current intensity (Table S2). Experiments were carried out within this wide range of light power. A constant area of the beam spot will become relevant in the finite element modeling (FEM) described below, when we determine the decrease in temperature as a function of the distance to the beam center.

The only two products observed in all of the experiments were CO and CH_4_, yet their selectivity was clearly influenced by incident light intensity. It should also be noted that a somewhat linear dependence between incident light power and local temperature reached was measured, hence resulting in reaction temperatures at the focal point ranging from 397 to 737 °C measured with a thermocouple in contact with the bed, without any external heat source, just by illumination for the lowest (3.97 W·cm^–2^) and highest (9.86 W·cm^–2^) employed light intensities, respectively (Table S2). Such high measured photoinduced temperatures can be attributed to a combination of high absorptivity, high illumination intensity, and low effective thermal conductivity (see discussion in the Section 3.3 and the Supporting Information). At the highest irradiation intensity, a CO_2_ conversion during the continuous flow experiments of about 41% was achieved. It is of great interest to comment on the change in product selectivity with an increasing irradiation power, methane being the major product at low light intensities, while CO is highly favored as the light intensity and, consequently, the reaction temperature increases. This shift in product selectivity is observed also in dark experiments (Figure 2b) upon increase of the reaction temperature in the range from 200 to 600 °C and, therefore, is not specific for the photocatalytic reaction. Note that to correlate Figures 2a (photothermal) and 2b (thermal) data, a light power of 3.97, 5.03, and 8.08 corresponds according to Table S2 to temperatures of 395, 419, and 615 °C, respectively. Moreover, the occurrence of any change in the Ru chemical state after the photocatalytic reactions was ruled out by acquiring the high-resolution Ru 3p + Ti 2p XPS zone after the reaction at 9.86 W·cm^–2^. The spectrum is shown in Figure S2 in the Supporting Information. As can be seen there, negligible changes in the Ru oxidation state were determined for the used photocatalyst in comparison to the fresh sample.

In order to assess the effect of light on the herein employed Ru/STO photocatalyst, different tests were carried out by removing from the solar spectrum different wavelength contributions via cutoff filters of different wavelength ranges normalized by the power supplied to the initial Xe source (Figure 2c). An additional test was also carried out by employing exclusively UV contribution (bandpass filter; 350 < λ < 380 nm). Recorded irradiation intensities under these conditions are shown in Table S3. Interestingly, under UV irradiation, no product evolution could be detected, ruling out a photochemical effect due to SrTiO_3_ band gap excitation. This lack of contribution of the UV region was complemented by blank controls using the full Xe output at 9.86 W cm^–2^ employing SrTiO_3_ as the photocatalyst, whereby no CO_2_ conversion was observed. To further stress a pure photothermal mechanism without any notable contribution from photogenerated charge separation processes arising from the photoexcitation of SrTiO_3_, we prepared under identical synthetic conditions a Ru/Al_2_O_3_ analogue. Al_2_O_3_ being an insulator oxide, its selection as a support was made to exclude any photoinduced charge separation contribution to the photothermal process. This obtained analogue was routinely characterized via PXRD, TEM, and STEM (Figures S3–S5). Acquired data points toward Ru NPs supported uniformly on the Al_2_O_3_ support, with particle sizes averaging 1.5 nm, comparable to that of Ru/SrTiO_3_, therefore excluding any possible influence arising from Ru NP geometry and size during catalytic output. Both materials, tested under identical conditions (Figure S6), show similar catalytic performance as well as final product distribution, therefore indicating a minimal effect of support choice. Moreover, nanosecond transient absorption spectroscopy (TAS) measurements were carried out in order to determine possible generation photoinduced charge separation in Ru/SrTiO_3_ and Ru/Al_2_O_3_ samples (Figure S7). The results show negligible transient signals on the microsecond time scale for any of the samples. Therefore, the photoinduced charge separation in these samples can be ruled out, or even if this takes place, the photogenerated charges recombine faster than our instrument response (50 ns), making unlikely the occurrence of any photochemical CO_2_conversion, due to very different time scales of the photoinduced charges (sub ns) and the reaction kinetics (micro- or millisecond time scale).

Furthermore, when employing the full visible spectrum after cutting the light contribution of wavelengths shorter than 360 nm, not much difference could be observed in comparison with the standard full solar irradiation test. In both cases, rates of around 1510 and 130 mmol·g^–1^·h^–1^·W^1–^cm^–2^ could be observed for CO and CH_4_, respectively. This lack of contribution of the UV region was complemented by blank controls using the full Xe output at 9.86 W·cm^–2^ employing SrTiO_3_ as photocatalyst, whereby no CO_2_ conversion was observed. All acquired data exclude the direct involvement of the SrTiO_3_ in photocatalytic mechanism, pointing toward a photothermal process driven solely by photon thermalization at the Ru NPs as active sites without contribution of photoinduced charge-separated states. In this purely photothermal mechanism, the role of STO would be mainly as thermal insulator support of Ru NPs. These Ru NPs would absorb photons and convert their energy into heat, which occurs across the whole visible range, as seen in Figure 2c. Normalizing the production rates by light intensity, some increase in the rates of the light-assisted CO_2_ hydrogenation was observed toward the NIR region, which is in agreement with the higher absorptivity of Ru/SrTiO_3_ in these longer wavelengths. To be noted is that upon irradiation at wavelengths longer than 455 nm, CH_4_ becomes the major product, an effect that can be associated with a weaker light intensity (Table S3) as an effect of employed cutoff filters, and thus a lower local temperature.

Photocatalyst stability was assessed for a long run of 96 h (Figure 3), observing some decrease in conversion in the first 20 h from 47 to about 42%, after which the reaction system maintains notable stability up to the latest measurement of about 100 h under continuous flow (44% CO_2_ conversion). It is proposed that the initial decay in activity is not directly related to photocatalyst deactivation but rather to the influence of a constant concentration of H_2_O in the system. Water is one of the reaction products and its influence is known to play an adverse role in the photothermal reaction, decreasing the CO_2_ conversion rate.^26^ Since water is not in the initial feed and its concentration on the photocatalyst grows on the initial reaction times, there could be a competition between the H_2_O molecules produced and intermediates such as HCOO*/HOCO*, causing an apparent decrease in photocatalyst activity at shorter times on stream. The evolution of water during catalytic output is illustrated in Figure S8. Photocatalyst stability after a long time of irradiation was also addressed via XRD and HRTEM characterization (Figures S9 and S10, respectively) of two Ru/STO samples after 4 and 96 h of photocatalytic operation. While no changes in XRD patterns were observed, indicating that STO crystallinity has been preserved, TEM images show an increase in Ru particle size from very small nanoparticles around 1 nm for the fresh photocatalyst to around 1.9 nm with agglomerates reaching up to 4 nm in the case of the photocatalyst subjected to 96 h of continuous irradiation (histogram illustrated in Figure S11). Upon analyzing the interplanar distance associated with the therein exposed crystallographic facet of the Ru particles that underwent some growth, a *d*-spacing of 2.3 Å was recorded. According to crystallographic databases (PDF #06–0663) this corresponds to the (100) facet of Ru^0^. This result is also in good agreement with the XPS data presented in Figure S2.

**Figure 3:**
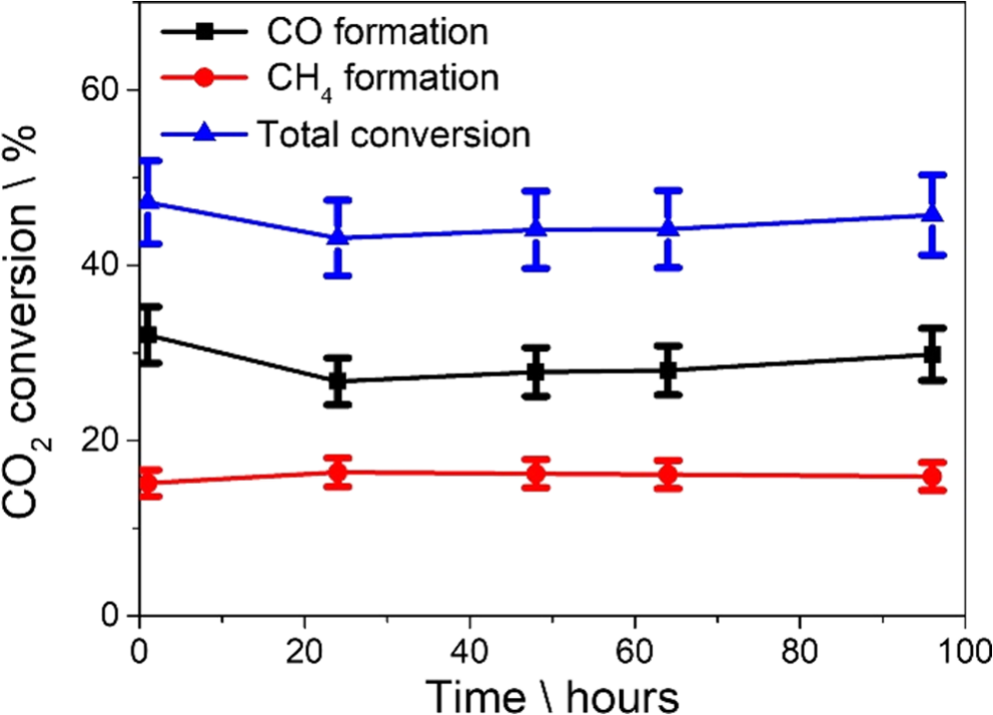
Catalytic output of the Ru/STO photocatalyst under 96 h of continuous irradiation (9.86 W·cm^–2^) and gas feed; CO formation (black squares), CH_4_ formation (red circles), and total conversion (blue triangles) are all illustrated for different reaction times. Lines have been drawn by interpolation of the experimental points. Reaction conditions: CO_2_/H_2_ ratio 1:1, gas flow 26 mL min^–1^, Ru/STO amount 100 mg, and light intensity as indicated in the axis.

This particle size growth is not unexpected. According to the literature, this is one of the most common reasons for catalyst aging.^33,34^ In any case, even though Ru NP size evolves during 96 h of irradiation, it has to be emphasized that the catalytic productivity remains notably constant, implying that the particle size range observed by TEM is of negligible influence in terms of catalytic output.

Rationalizing the change in product selectivity. One of the main observations of the present study is the experimental evidence of product selectivity shift under an increasing light irradiation intensity and hence an increase in local reaction temperatures. This selectivity shift from CH_4_ to CO observed in the photothermal reactions follows the trend also observed for the pure thermal reactions as shown in Figure 2b. To understand this selectivity change and to provide some evidence supporting the assessment adopted herein, several light-assisted catalytic experiments were carried out. Photocatalytic CO_2_ hydrogenation using the Ru/STO photocatalyst was carried out under different light intensities in batch conditions. The CO_2_ and H_2_ product evolution was followed over time, and the results for the case of 9.86 W·cm^–2^ power are presented in Figures 4 and S12, respectively. As can be seen there, both products, CH_4_ and CO appear at the initial time, meaning that both are primary products. However, methane selectivity was much higher at initial times and reached a maximum selectivity that decreased at longer reaction times. In comparison, CO temporal evolution shows a continuous increase in selectivity. These temporal profiles indicate that CH_4_ percentage decreases from 22% at 15 h to 19% at 90 h. This stationary CH_4_ value while CO formation continues growing indicates a balance between CH_4_ formation from CO_2_ and CH_4_ decomposition to CO. CO appears as the primary and secondary stable product. To provide support to the lack of CH_4_ stability further tests were carried out considering that there are two possible reactions for methane conversion, either the reaction with water (steam reforming) or the reaction with CO_2_ (dry reforming). To check the occurrence of methane conversion by reaction with water or CO_2_, two separate independent experiments were carried out by filling the reactor with methane and water vapor or methane and CO_2_. While water was introduced in the photoreactor in excess, the molar ratio between CH_4_ and CO_2_ was 1-to-1. The reactions were performed at the maximum irradiation power (9.86 W·cm^–2^) with simulated sunlight. In both experiments, CO evolution was recorded, with a slightly faster initial reaction rate (*t* < 50 min) for steam reforming in comparison with dry reforming. Even at lower light intensities of 6.9 and 8.39 W·cm^–2^ appearance of CO in the CH_4_ steam and dry reforming was also observed, although in lesser proportions as shown in Figure 4b. This is in accordance with the relative temperature that is lower as the light intensity decreases. Therefore, it can be concluded that at a high light intensity, as CH_4_ is being formed in the presence of CO_2_ and water, it decomposes by both steam and dry reforming (eqs 1 and 2) becoming converted into CO and H_2_. In the case of dry reforming, disappearance of CH_4_ and CO_2_ was according to eq 2. These two established catalytic reactions^35,36^ explain well the change in the product distribution observed here and provide an understanding of the contrasting data reported in the literature for different reaction conditions.

1 $${\mathrm{CH}}_{4}+H_{2}O\to \mathrm{CO}+3H_{2}$$

2 $${\mathrm{CH}}_{4}+{\mathrm{CO}}_{2}\to 2\mathrm{CO}+2H_{2}$$

**Figure 4:**
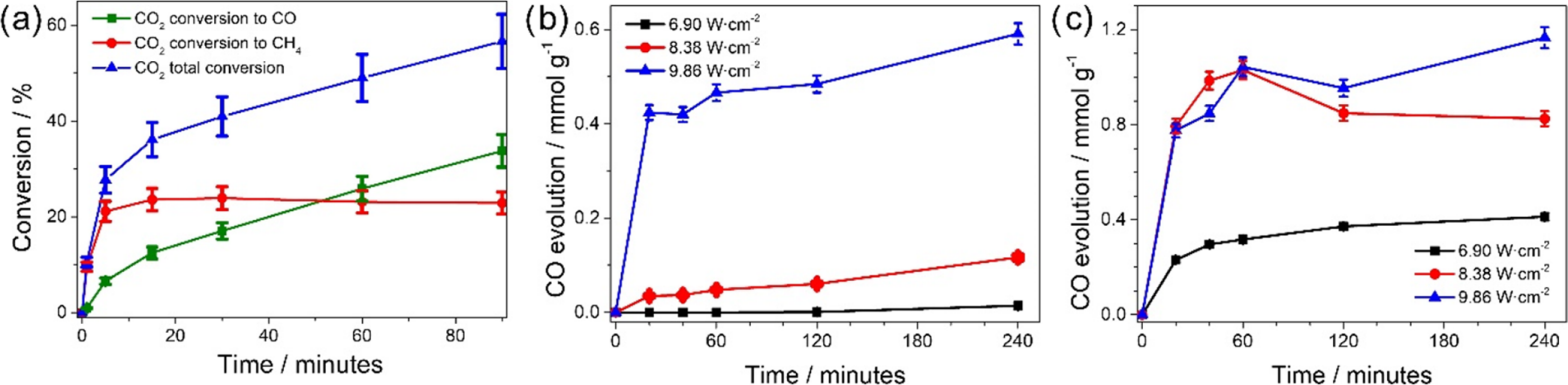
(a) Batch reaction carried out with a 9.86 W·cm^–2^ power irradiation of CO_2_/H_2_ mixtures of 1:1 v/v; conversion of CO_2_ to CO (green squares), conversion of CO_2_ to CH_4_ (red circles) and total CO_2_ conversion (blue triangle). (b) Steam reforming reaction carried out at different light intensity (black squares, red dots, and blue triangles for 6.90, 8.38, and 9.86 W·cm^–2^, respectively) in batch, 20 vol % CH_4_ in Ar and 100 μL of water added prior to irradiation. (c) Batch dry reforming reaction carried out at different light irradiation intensities (black squares, red dots, and blue triangles for 6.90, 8.38, and 9.86 W cm^–2^, respectively), 20 vol % CH_4_ and 20 vol % CO_2_ in Ar.

### 3.3 Finite Element Modeling

To obtain insight into the *operando* temperature distribution around the catalyst pellet, the photoreactor (see Figure S13) was modeled using the FEM method in COMSOL Multiphysics (see the Supporting Information for details). Briefly, a coupled heat transfer and fluid dynamics simulation was set up to fully account for the light-driven heating, gas flow, and heat losses through conduction and convection. Light-induced heat generation was approximated as a two-dimensional heat source on the top surface of the pellet because it was assumed that all light is absorbed or diffuse-reflected at the top few μm as a result of the highly scattering nature of the nanoparticulate solid. Since the catalyst pellet consists of loosely bound NPs, the effective thermal conductivity of the catalyst pellet was close to that of the CO_2_/H_2_ gas mixture.^37^

The results show that focused sunlight irradiation of the catalyst pellet induces large thermal gradients inside the material and convective gas flows inside the reactor (Figure 5). For an illumination intensity of 9.86 W·cm^–2^, the top-center of the catalyst pellet reaches a temperature of 900 K (Figure 5a), while the (unilluminated) bottom-center of the catalyst remains at 445 K and the top-surface edges of the catalyst at 370 K. These large temperature gradients are a direct unavoidable consequence of (1) the Gaussian energy profile of the focused light beam, which maximizes the energy at the focal point; (2) the fact that the optical irradiation is approximated to a two-dimensional heat source on the top surface, due to the negligible light penetration depth in the highly scattering and absorbing solid; and (3) the very low effective thermal conductivity of the photocatalyst that minimizes thermal equilibration. The large temperature gradient in the gas phase drives convective flows in both the top and bottom compartments of the photoreactor, reaching a peak velocity of 0.2 m·s^–1^ above the illumination spot. For all experimentally tested light intensities, the same convection pattern is observed, and the catalyst temperature and gradients scale almost linearly with light intensity (Figures 5c and S14).

**Figure 5:**
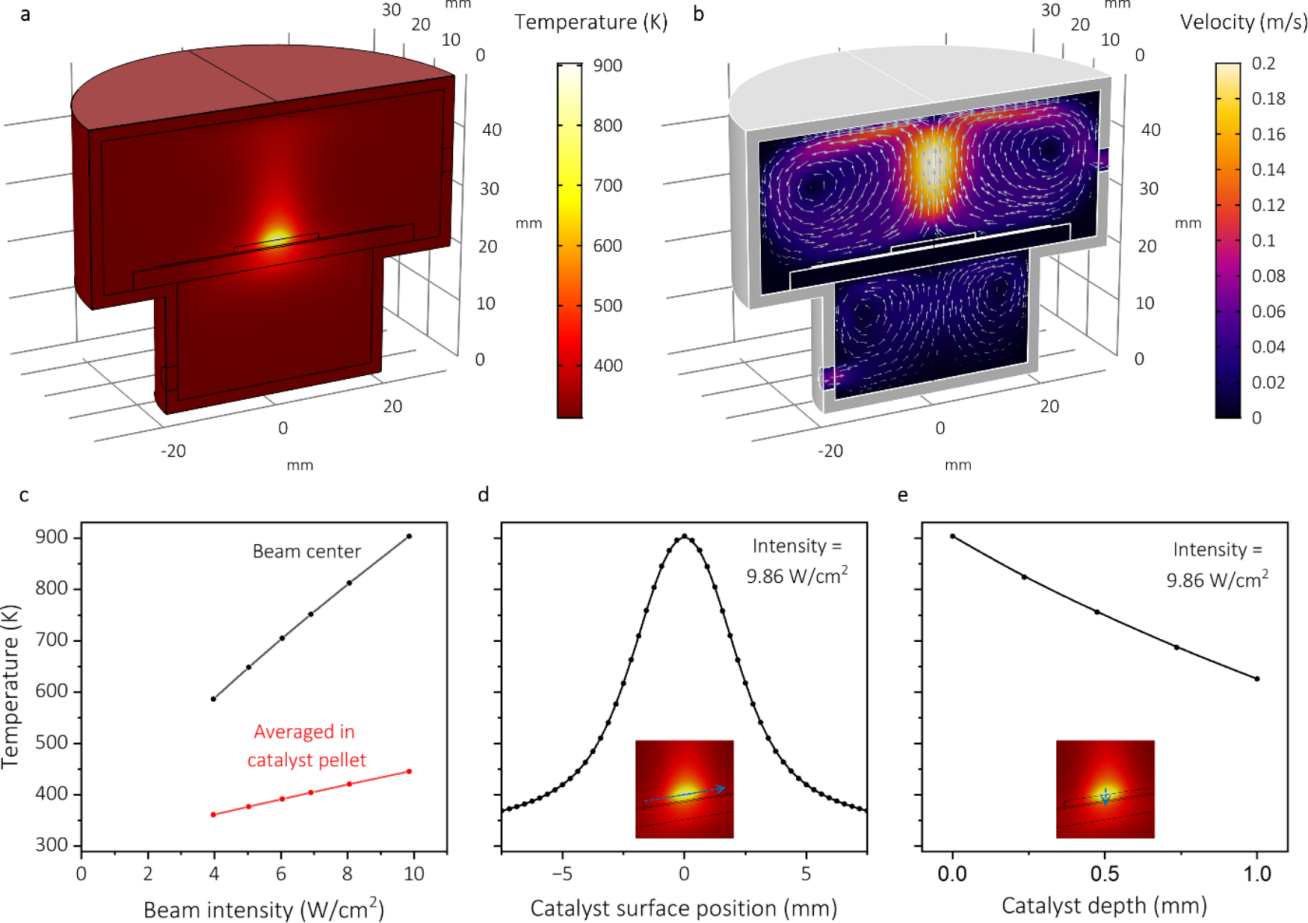
Finite element modeling of the photothermal reactor. (a) Temperature distribution under 9.86 W·cm^–2^ illumination. (b) Magnitude of the gas velocity. (c) Temperature of the catalyst pellet as a function of illumination intensity, at the beam center and catalyst top-surface (black) and as an averaged value of the entire catalyst pellet (red). (d) Temperature distribution along the catalyst top surface with respect to the beam center (i.e., along the blue line in the inset), for an illumination intensity of 9.86 W·cm^–2^. (e) Catalyst temperature as a function of depth at the beam center.

Within the FEM modeling, we have investigated the macroscopic temperature variations inside the catalyst pellets. To demonstrate that such a temperature is representative for the actual catalyst temperature and that no temperature variations exist at the NP scale, we approximate the system with a two-dimensional array of Ru NPs and calculate the local heating by the experimentally proven calculations of Baffou et al.^32^ In this fictional array, the Ru NPs are 3 nm in diameter and spaced 5 nm apart in a square lattice on an STO surface, which approaches the HRTEM images. Then, the local temperature of each Ru NP is the sum of the ambient temperature (*T*_amb_), light-induced heating by the nanoparticle itself (Δ*T*_self_), and the collective heating originating from adjacent particles (Δ*T*_coll_)^32,38^

3 $$T=T_{\mathrm{amb}}+\Delta T_{\mathrm{self}}+\Delta T_{\mathrm{coll}}$$

The self-heating term can be calculated from the absorption cross section of each Ru NP, estimated from Mie scatter calculations^39^ (σ_abs_ ≈ 1 × 10^–19^ m^2^ in the visible range, *i.e*., much smaller than the geometric cross section of 7 × 10^–18^ because the particles absorb light weakly), the illumination power (*P* = 2.47 W), the full width at half-maximum (fwhm) beam diameter (***H*** = 2.1 mm), the effective thermal conductivity (κ_eff_ ≈ 0.2 W/(m·K), see the Supporting Information), and the NP radius (*r* = 1.5 nm) according to eq 4.^32^

4 $$\Delta T_{\mathrm{self}}=\frac{\ln\left(2\right)\sigma_{\mathrm{abs}}P}{\pi^{2}H\kappa_{\mathrm{eff}}r}$$

For the highest illumination intensity (9.86 W·cm^–2^), Δ*T*_self_ amounts to only 5 × 10^–5^ K, because an individual and small Ru NP only absorbs weakly (σ_abs_ ≪ πr^2^). Further, for Gaussian beam illumination, the collective heating contribution at the center of the array is given by^32^

5 $$\Delta T_{\mathrm{coll}}=\frac{\sigma_{\mathrm{abs}}P}{\kappa_{\mathrm{eff}}}\sqrt{\frac{\ln\left(2\right)}{4\pi }}\frac{1}{\mathrm{HA}}\left(1-\frac{4\sqrt{\ln\left(2\right)A}}{\pi H}\right)$$

where ***A*** is the array unit cell area (2.5 × 10^–17^ m^2^ for 5 nm square lattice spacing) and Δ*T*_coll_ amounts to 22 K at 9.86 W·cm^–2^.

Thus, Δ*T*_coll_ ≫ Δ*T*_self_, which means that the catalyst temperature is dictated by the cumulative photothermal heating of all small Ru NPs combined, as they are very closely spaced, and that no temperature gradients exist between the Ru NPs and their immediate STO/gas surroundings. This difference between self-heating and collective heating becomes even greater when considering that the actual experimental catalyst is not a two-dimensional, but a three-dimensional array of Ru NPs.^40^ Based on these calculations, we conclude that the FEM simulations offer an accurate approximation of both the macroscopic and microscopic temperatures within the catalyst porous solid.

## 4 Conclusions

The present results show a shift in product selectivity from methane (low irradiation intensity) to CO (high irradiation intensity) for the same Ru/STO catalyst as a function of light intensity. These data indicate that the previously contrasting methane or CO selectivity reported in the literature for similar photocatalysts is most likely due to the dependency of methane stability under different reaction conditions. By performing independent photocatalytic irradiation with varied light intensities, in the presence of water and CO_2_ excess, it has been observed that methane reacts photocatalytically by steam reforming (more favorable, somewhat higher reaction rates) and dry reforming. The product in these two reactions is CO that was found to be stable under the reaction conditions. This behavior is exactly what can be expected in purely thermal catalysis in which CO selectivity increases with temperature^41–43^ and CH_4_ steam or dry reforming requires even higher temperatures. In addition, finite element modeling has shown that for these powdered Ru catalysts having a high density of NPs on the support surface, the bulk temperature at high light intensity reflects well the temperature at the NP surface, reaching very high temperatures over 600 °C at the center of the illuminated focused spot and diminishing considerably upon moving a few millimeters with the lateral distance or depth. Therefore, the present study provides a definite clarification of the role of light intensity from one sun to about 100 suns on the photothermal CO_2_ hydrogenation.

## Supplementary Material



## Notes

Catalyst preparation and characterization details, photothermal reactions both in flow and in batch conditions, photothermal steam and dry reforming, thermal CO_2_ hydrogenation, product quantification, table summarizing reported photothermal CO_2_ hydrogenation data, finite element modeling details, diffuse reflectance measurements, table with macroscopic temperature as a function of the simulated sunlight power, table with the light power as a function of the cutoff filter, figures with Ru NP size distribution for fresh sample and after 4 and 96 h irradiation, water evolution upon irradiation, XRD comparison for the fresh and 100 h used Ru/STO, HRTEM and ADF-STEM images of Ru/STO after 4 and 96 h irradiation, schematics of the photothermal reactor, temperature distribution, and gas velocity inside the illuminated photoreactor, and setup for diffuse reflectance measurements (PDF)

## References

[1] Peters G. P., Andrew R. M., Boden T., Canadell J. G., Ciais P., Le Quéré C., Marland G., Raupach M. R., Wilson C. (2013). The challenge to keep global warming below 2 °C. Nat. Clim. Change.

[2] Mac
Dowell N., Fennell P. S., Shah N., Maitland G. C. (2017). The role
of CO2 capture and utilization in mitigating climate change. Nat. Clim. Change.

[3] Peng Y., Szalad H., Nikacevic P., Gorni G., Goberna S., Simonelli L., Albero J., López N., García H. (2023). Co-doped hydroxyapatite
as photothermal catalyst for
selective CO2 hydrogenation. Appl. Catal., B.

[4] Galhardo T. S., Braga A. H., Arpini B. H., Szanyi J., Gonçalves R. V., Zornio B. F., Miranda C. R., Rossi L. M. (2021). Optimizing Active
Sites for High CO Selectivity during CO2 Hydrogenation over Supported
Nickel Catalysts. J. Am. Chem. Soc..

[5] Wang L., Dong Y., Yan T., Hu Z., Ali F. M., Meira D. M., Duchesne P. N., Loh J. Y. Y., Qiu C., Storey E. E. (2020). Black
indium oxide a photothermal CO2 hydrogenation
catalyst. Nat. Commun..

[6] Zhang J., Yang Y., Liu J., Xiong B. (2021). Mechanistic understanding
of CO2 hydrogenation to methane over Ni/CeO2 catalyst. Appl. Surf. Sci..

[7] Zhou G., Liu H., Xing Y., Xu S., Xie H., Xiong K. (2018). CO2 hydrogenation
to methane over mesoporous Co/SiO2 catalysts: Effect of structure. J. CO2 Util..

[8] Mateo D., Morlanes N., Maity P., Shterk G., Mohammed O. F., Gascon J. (2021). Photothermal Catalysis: Efficient
Visible-Light Driven
Photothermal Conversion of CO2 to Methane by Nickel Nanoparticles
Supported on Barium Titanate. Adv. Funct. Mater..

[9] Dreyer J. A. H., Li P., Zhang L., Beh G. K., Zhang R., Sit P. H. L., Teoh W. Y. (2017). Influence of the oxide support reducibility
on the CO2 methanation over Ru-based catalysts. Appl. Catal., B.

[10] Mateo D., Albero J., García H. (2019). Titanium-Perovskite-Supported
RuO2
Nanoparticles for Photocatalytic CO2Methanation. Joule.

[11] Kattel S., Ramírez P. J., Chen J. G., Rodriguez J. A., Liu P. (2017). Active sites for CO2 hydrogenation to methanol on Cu/ZnO catalysts. Science.

[12] Khan M. U., Wang L., Liu Z., Gao Z., Wang S., Li H., Zhang W., Wang M., Wang Z., Ma C., Zeng J. (2016). Pt3Co Octapods as Superior
Catalysts of CO2 Hydrogenation. Angew. Chem.,
Int. Ed..

[13] Witoon T., Chalorngtham J., Dumrongbunditkul P., Chareonpanich M., Limtrakul J. (2016). CO2 hydrogenation to methanol over Cu/ZrO2 catalysts:
Effects of zirconia phases. Chem. Eng. J..

[14] Rui N., Wang Z., Sun K., Ye J., Ge Q., Liu C.-j. (2017). CO2 hydrogenation to methanol over
Pd/In2O3: effects
of Pd and oxygen vacancy. Appl. Catal., B.

[15] Albrecht M., Rodemerck U., Schneider M., Bröring M., Baabe D., Kondratenko E. V. (2017). Unexpectedly
efficient CO2 hydrogenation
to higher hydrocarbons over non-doped Fe2O3. Appl. Catal., B.

[16] Chen G., Gao R., Zhao Y., Li Z., Waterhouse G. I. N., Shi R., Zhao J., Zhang M., Shang L., Sheng G. (2018). Alumina-Supported CoFe
Alloy Catalysts Derived from
Layered-Double-Hydroxide Nanosheets for Efficient Photothermal CO2
Hydrogenation to Hydrocarbons. Adv. Mater..

[17] De
la Rosa-Priego F. A., Gutierrez-López E. D., Zepeda T. A., Acosta-Alejandro M., Venezia A. M., Fuentes-Moyado S., Pawelec B., Díaz-de-León J. N. (2021). Enhanced CO2 Hydrogenation
to C2+ Hydrocarbons over Mesoporous x%Fe2O3–Al2O3 Catalysts. Ind. Eng. Chem. Res..

[18] Jeantelot G., Følkner S. P., Manegold J. I. S., Ingebrigtsen M. G., Jensen V. R., Le Roux E. (2022). Selective
Hydrodeoxygenation of Lignin-Derived
Phenols to Aromatics Catalyzed by Nb2O5-Supported Iridium. ACS Omega.

[19] Wei J., Yao R., Ge Q., Xu D., Fang C., Zhang J., Xu H., Sun J. (2021). Precisely
regulating Brønsted acid sites to promote
the synthesis of light aromatics via CO2 hydrogenation. Appl. Catal., B.

[20] Nezam I., Zhou W., Gusmão G. S., Realff M. J., Wang Y., Medford A. J., Jones C. W. (2021). Direct
aromatization of CO2 via combined
CO2 hydrogenation and zeolite-based acid catalysis. J. CO2 Util..

[21] Olah G. A., Mathew T., Prakash G. K. S. (2017). Chemical Formation
of Methanol and
Hydrocarbon (“Organic”) Derivatives from CO2 and H2—Carbon
Sources for Subsequent Biological Cell Evolution and Life’s
Origin. J. Am. Chem. Soc..

[22] Fan W. K., Tahir M. (2022). Recent developments
in photothermal reactors with understanding on
the role of light/heat for CO2 hydrogenation to fuels: A review. Chem. Eng. J..

[23] Zhao J., Bai Y., Liang X., Wang T., Wang C. (2021). Photothermal catalytic
CO2 hydrogenation over molybdenum carbides: Crystal structure and
photothermocatalytic synergistic effects. J.
CO2 Util..

[24] Cai M., Wu Z., Li Z., Wang L., Sun W., Tountas A. A., Li C., Wang S., Feng K., Xu A.-B. (2021). Greenhouse-inspired
supra-photothermal CO2 catalysis. Nat. Energy.

[25] Szalad H., Peng L., Primo A., Albero J., García H. (2021). Fe clusters
embedded on N-doped graphene as a photothermal catalyst for selective
CO2 hydrogenation. Chem. Commun..

[26] Mateo D., Albero J., García H. (2018). Graphene supported
NiO/Ni nanoparticles
as efficient photocatalyst for gas phase CO2 reduction with hydrogen. Appl. Catal., B.

[27] Wang S., Zhang D., Wang W., Zhong J., Feng K., Wu Z., Du B., He J., Li Z., He L. (2022). Grave-to-cradle upcycling of Ni from electroplating
wastewater to
photothermal CO2 catalysis. Nat. Commun..

[28] Li Z., Liu J., Shi R., Waterhouse G. I. N., Wen X.-D., Zhang T. (2021). Fe-Based Catalysts
for the Direct Photohydrogenation of CO2 to Value-Added Hydrocarbons. Adv. Energy Mater..

[29] Mateo D., Cerrillo J. L., Durini S., Gascon J. (2021). Fundamentals and applications
of photo-thermal catalysis. Chem. Soc. Rev..

[30] Wu Z., Shen J., Li C., Zhang C., Feng K., Wang Z., Wang X., Meira D. M., Cai M., Zhang D. (2023). Mo2TiC2MXene-Supported
Ru Clusters for Efficient Photothermal
Reverse Water–Gas Shift. ACS Nano.

[31] Hogan N. J., Urban A. S., Ayala-Orozco C., Pimpinelli A., Nordlander P., Halas N. J. (2014). Nanoparticles Heat through Light
Localization. Nano Lett..

[32] Baffou G., Berto P., Ureña E. B., Quidant R., Monneret S., Polleux J., Rigneault H. (2013). Photoinduced
Heating of Nanoparticle
Arrays. ACS Nano.

[33] Dai Y., Lu P., Cao Z., Campbell C. T., Xia Y. (2018). The physical chemistry
and materials science behind sinter-resistant catalysts. Chem. Soc. Rev..

[34] Hansen T. W., DeLaRiva A. T., Challa S. R., Datye A. K. (2013). Sintering of Catalytic
Nanoparticles: Particle Migration or Ostwald Ripening?. Acc. Chem. Res..

[35] Sheu E. J., Mokheimer E. M. A., Ghoniem A. F. (2015). A review of solar methane reforming
systems. Int. J. Hydrogen Energy.

[36] Li D., Nakagawa Y., Tomishige K. (2011). Methane reforming to synthesis gas
over Ni catalysts modified with noble metals. Appl. Catal., A.

[37] Un I. W., Dubi Y., Sivan Y. (2022). Photothermal nonlinearity
in plasmon-assisted
photocatalysis. Nanoscale.

[38] Maibohm C., Brewer J. R., Sturm H., Balzer F., Rubahn H.-G. (2006). Bleaching
and coating of organic nanofibers. J. Appl.
Phys..

[39] Bohren, C. F. ; Huffman, D. R. Angular Dependence of Scattering. In Absorption and Scattering of Light by Small Particles; Wiley, 1998; pp 381–428.

[40] Naef, A. ; Tsoulos, T. V. ; Tagliabue, G. Photothermal and Thermo-optical Effects in 3D Arrays of Dielectric and Plasmonic Nanoantennas. 2022, arXiv:2211.11832v1. arXiv.org e-Print archive. 10.48550/arXiv.2211.11832.

[41] Kwak J. H., Kovarik L., Szanyi J. (2013). CO2 Reduction
on Supported Ru/Al2O3
Catalysts: Cluster Size Dependence of Product Selectivity. ACS Catal..

[42] Tada S., Ochieng O. J., Kikuchi R., Haneda T., Kameyama H. (2014). Promotion
of CO2 methanation activity and CH4 selectivity at low temperatures
over Ru/CeO2/Al2O3 catalysts. Int. J. Hydrogen
Energy.

[43] González-Castaño M., Dorneanu B., Arellano-García H. (2021). The reverse water gas
shift reaction: a process systems engineering perspective. React. Chem. Eng..

