# Supplementary material for: Solving the Conundrum of the Influence of Irradiation Power on Photothermal CO2 Hydrogenation
Source: ACS Catal. 2025 Feb 19;15(5):3836–45. doi: 10.1021/acscatal.5c00247 (PMC12123606; doi:10.1021/acscatal.5c00247)
Supplement: Supplementary file 1 [file cs5c00247_si_001.pdf]

Supporting Information

Solving the Conundrum of the Influence of Irradiation Power on Photothermal CO_2 Hydrogenation

Horatiu Szalad[1], Yong Peng[1], Jonas Werner Gosch[1], Andrea Baldi[2], Sven H. C. Askes[2]*, Josep Albero[1]* and Hermenegildo García[1]*

[1]Instituto Universitario de Tecnología Química (CSIC-UPV, Universitat Politècnica de València, Avda. de los Naranjos s/n, 46022, Valencia, Spain.

[2]Department of Physics and Astronomy, Vrije Universiteit Amsterdam, De Boelelaan 1081, 1081 HV Amsterdam, Netherlands

Corresponding Author

Sven H.C. Askes – Department of Physics and Astronomy, Vrije Universiteit Amsterdam, De Boelelaan 1081, 1081 HV Amsterdam, Netherlands; ORCID: 0000-0001-6538-3645; E-mail: s.h.c.askes@vu.nl.

Josep Albero – Instituto Universitario de Tecnología Química (CSIC-UPV), Universitat Politècnica de València, Avda. de los Naranjos s/n, 46022, Valencia, Spain; ORCID: 0000-0002-4841-7206; E-mail:joalsan6@itq.upv.es

Hermenegildo García – Instituto Universitario de Tecnología Química (CSIC-UPV), Universitat Politècnica de València, Avda. de los Naranjos s/n, 46022, Valencia, Spain; ORCID: 0000-0002-9664-493X; E-mail:hgarcia@qim.upv.es

Table S1. Literature data for Ru based photothermal catalysts reporting different product and selectivity, either CH_4 or CO, depending on irradiation power/reaction temperature.

**Reported Catalyst**	**Irradiation Source**	**External heating**	**CO_2:H_2 v/v ratio**	**CO selectivity**	**CH_4 selectivity**	**Reference**
Ru(2.5 wt.%)@STO	AM1.5G solar simulated light – 9.86 W·cm^{-2}	no	1:1	79%	21%	This work
Ru(2.5 wt.%)@STO	AM1.5G solar simulated light – 3.97 W·cm^{-2}	no	1:1	27%	73%	This work
RuO_2/STO	Xe lamp – 0.1 W·cm^{-2}	150°C	1:4	--	100%	Mateo et al. [2]
Ru/Mo_2TiC_2	Xe lamp – 3.8 W·cm^{-2}	no	1:1	99%	--	Wu et al. [3]
Ru-TiO_x	Xe lamp – 1 W·cm^{-2}	no	1:4	--	99%	Dong et al.[4]
Ru-8.3-150°C	Xe lamp – 1.8 W·cm^{-2}	no	1:1	46%	54%	Kong et al. [5]
Ru/SiO_2	Xe lamp – 35 mW·cm^{-2}	300°C	1:6	--	100%	Kim et al.[6]
Ru-Al_2O_3-x-L	Xe lamp – 2.27 W·cm^{-2}	no	1:4	--	99%	Liu et al.[7]

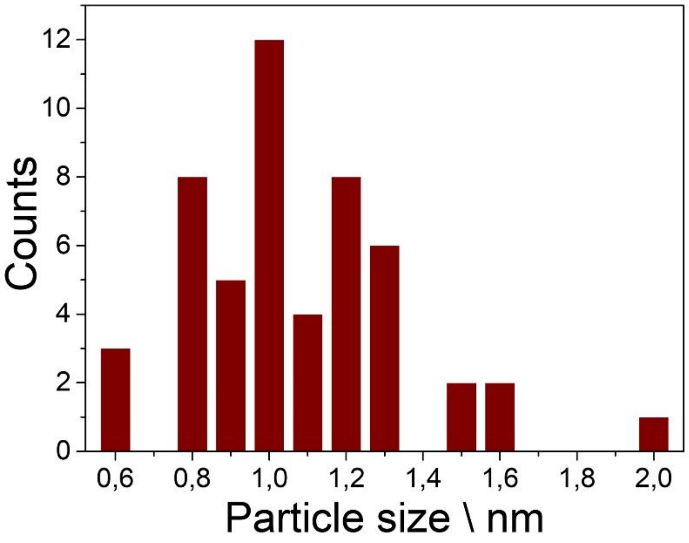

Figure S1. Histogram of Ru nanoparticle size distribution in Ru/STO.

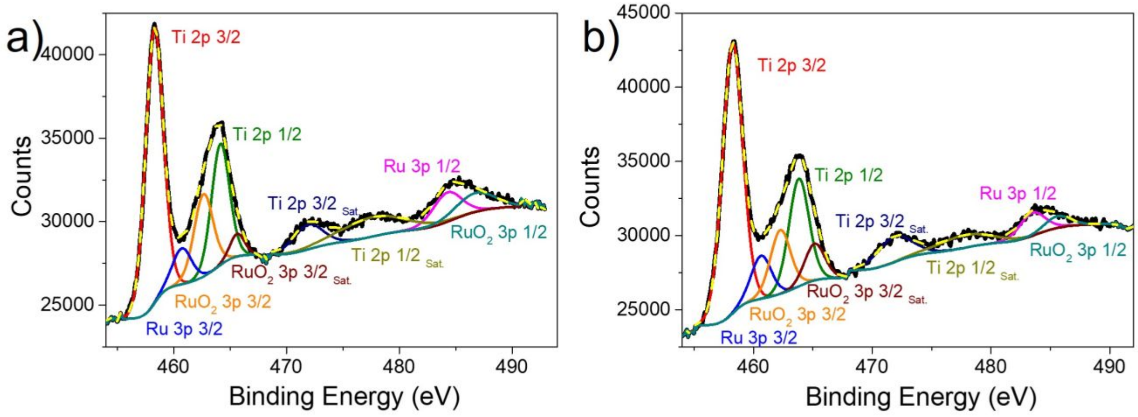

Figure S2. High resolution Ru 3p + Ti 2p XPS spectra of as-prepared Ru/STO (a) and after reaction at 9.86 W·cm^{-2} light intensity (b).

Table S2. Values of the recorded temperatures at each applied irradiation power.

**Light Intensity \ W·cm^{-2}**	**Temperature \ ^{o}C**
3.97	397
5.03	419
6.04	476
6.9	522
8.06	615
9.86	737

Table S3. Values of the recorded irradiation powers for different cut-off/bandpass filters.

**Employed Cut-off Filter**	**Measured Light Intensity (W·cm^{-2})**
N/A (only AM 1.5)	5.223
AM1.5 + 360nm	4.872
AM1.5 + 455nm	3.956
AM1.5 + 515nm	3.245
AM1.5 + 610nm	1.932

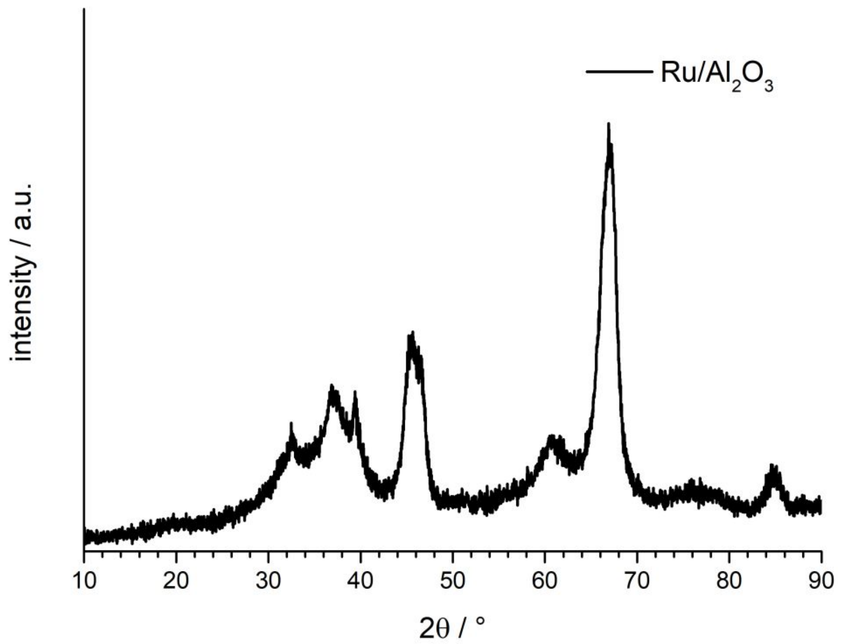

Figure S3. PXRD pattern recorded for Ru/Al_2O_3 analogue photocatalyst.

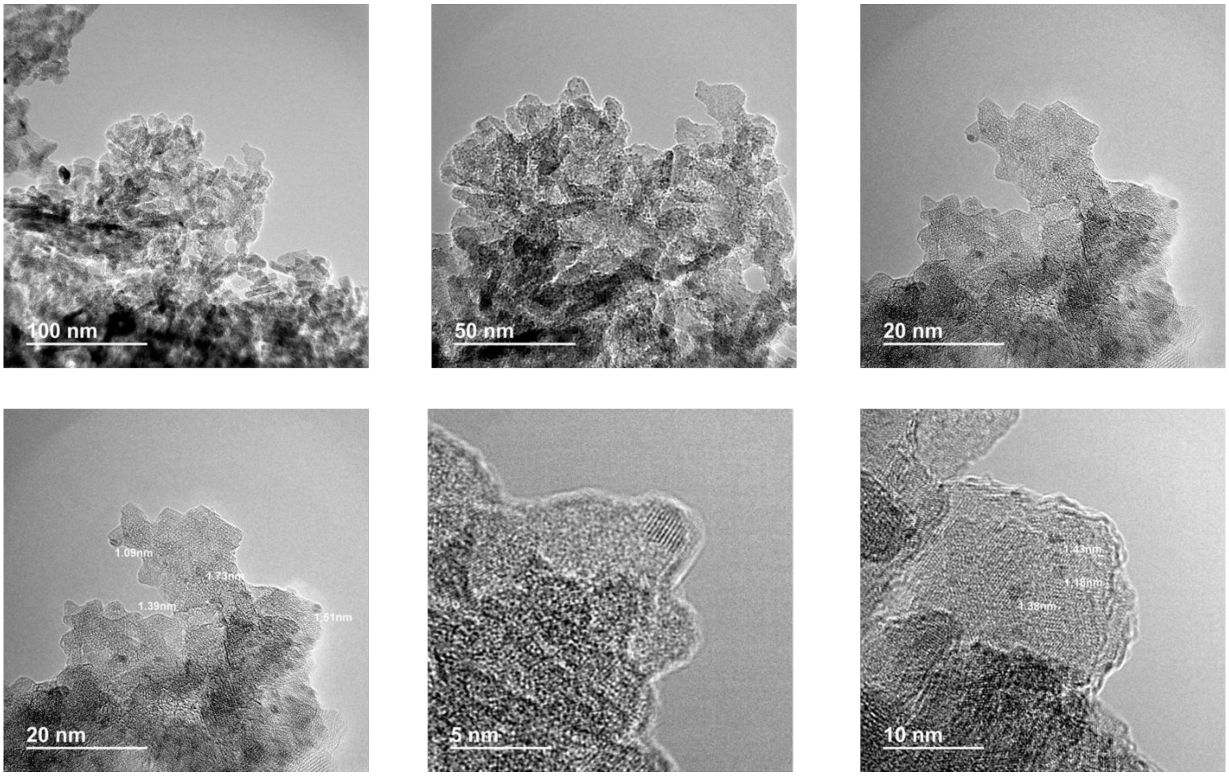

Figure S4. TEM images obtained for the Ru/Al_2O_3 analogue. Observed lattice fringes correspond to a RuO_2 phase. The size of isolated Ru particles is displayed in selected images.

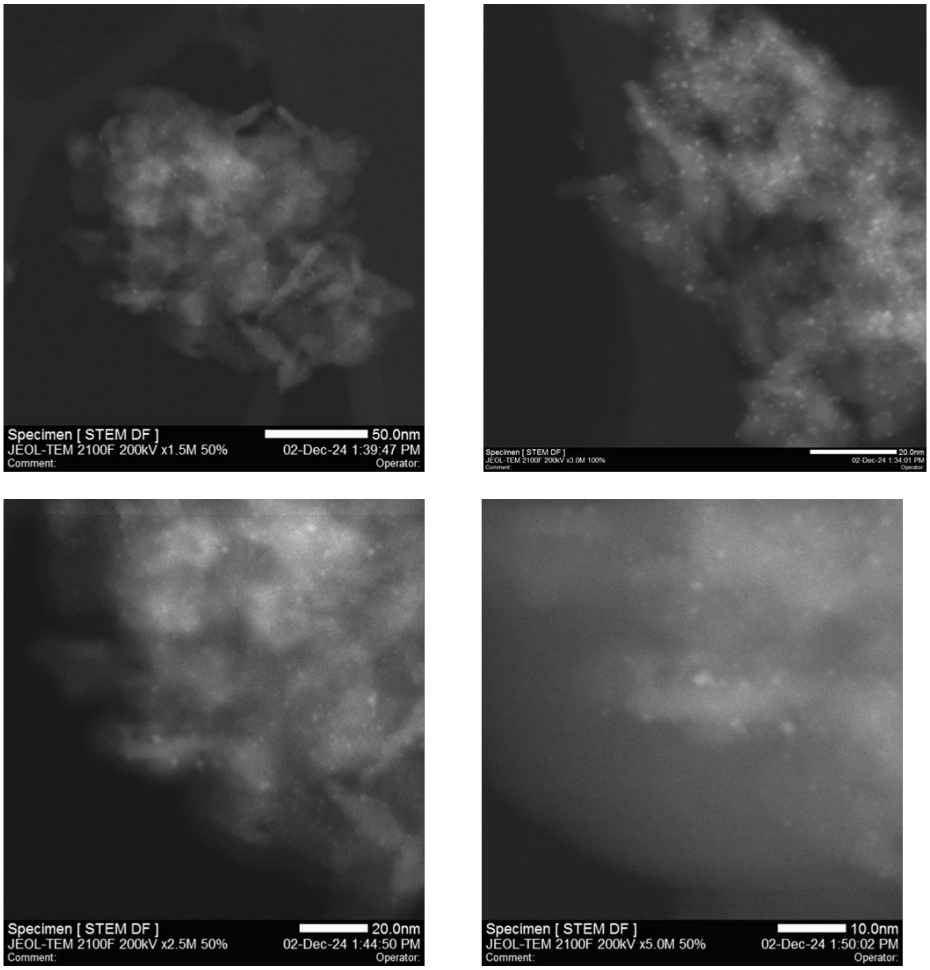

Figure S5. STEM dark field images recorded for the Ru/Al_2O_3 analogue. A uniform distribution of Ru NPs is evidenced.

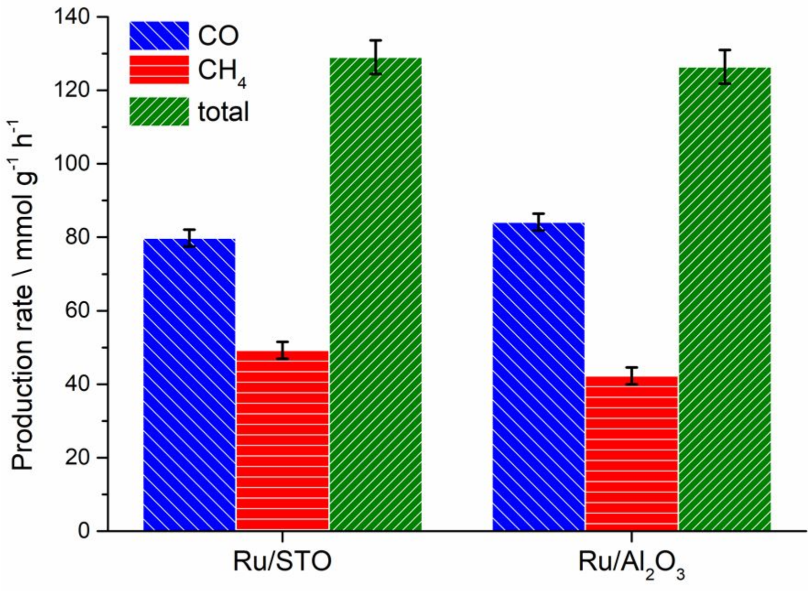

.

Figure S6. Catalytic performance of Ru/STO and Ru/Al_2O_3 under a 5.22 W·cm^2 light irradiation (AM 1.5). Corresponding reaction rates are illustrated with blue (CO), red (CH_4) and green (total CO_2 conversion) bars. Reaction conditions: CO_2/H_2 ratio 1:1, gas flow 26 mL min^{-1}, Ru/STO amount 100 mg.

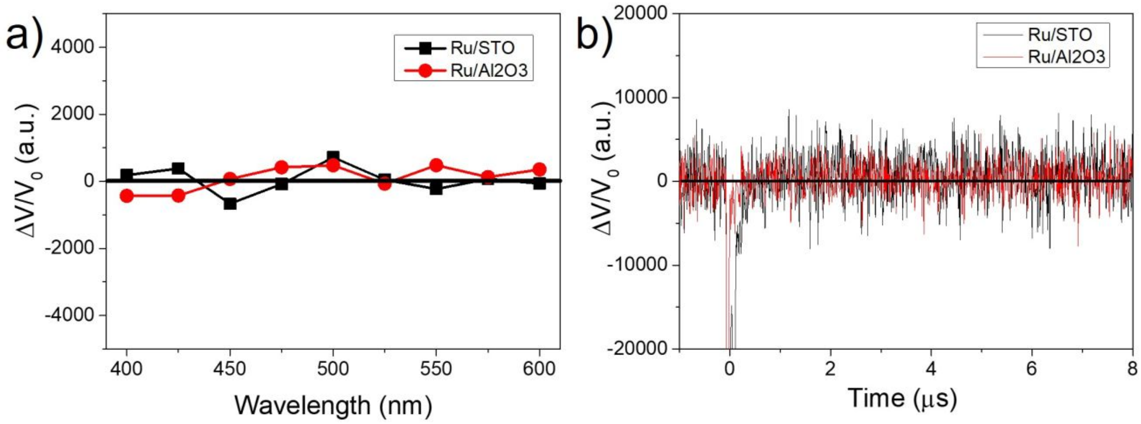

Figure S7. (a) Transient absorption spectra of N_2-saturated Ru/STO and Ru/Al_2O_3 dispersions in acetonitrile acquired at 1 µs. (b) Transient absorption decays of N_2-saturated Ru/STO and Ru/Al_2O_3 dispersions in acetonitrile monitored at 500 nm. Laser excitation 355 nm.

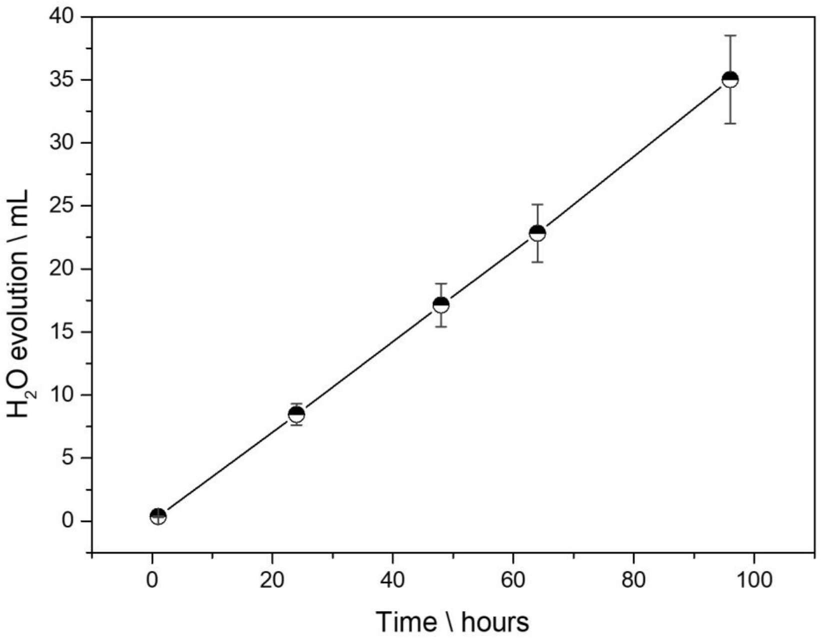

Figure S8. Water evolution during the 96 h robustness test.

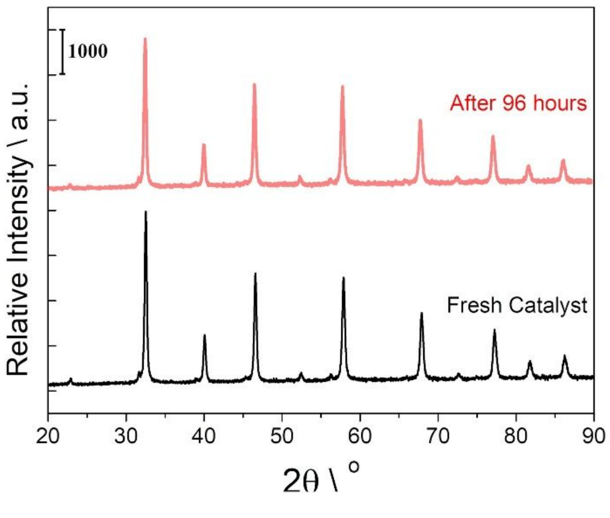

Figure S9. PXRD peaks acquired for the Ru/STO sample subjected to 96 h operation (red) in comparison with the fresh photocatalyst (black).

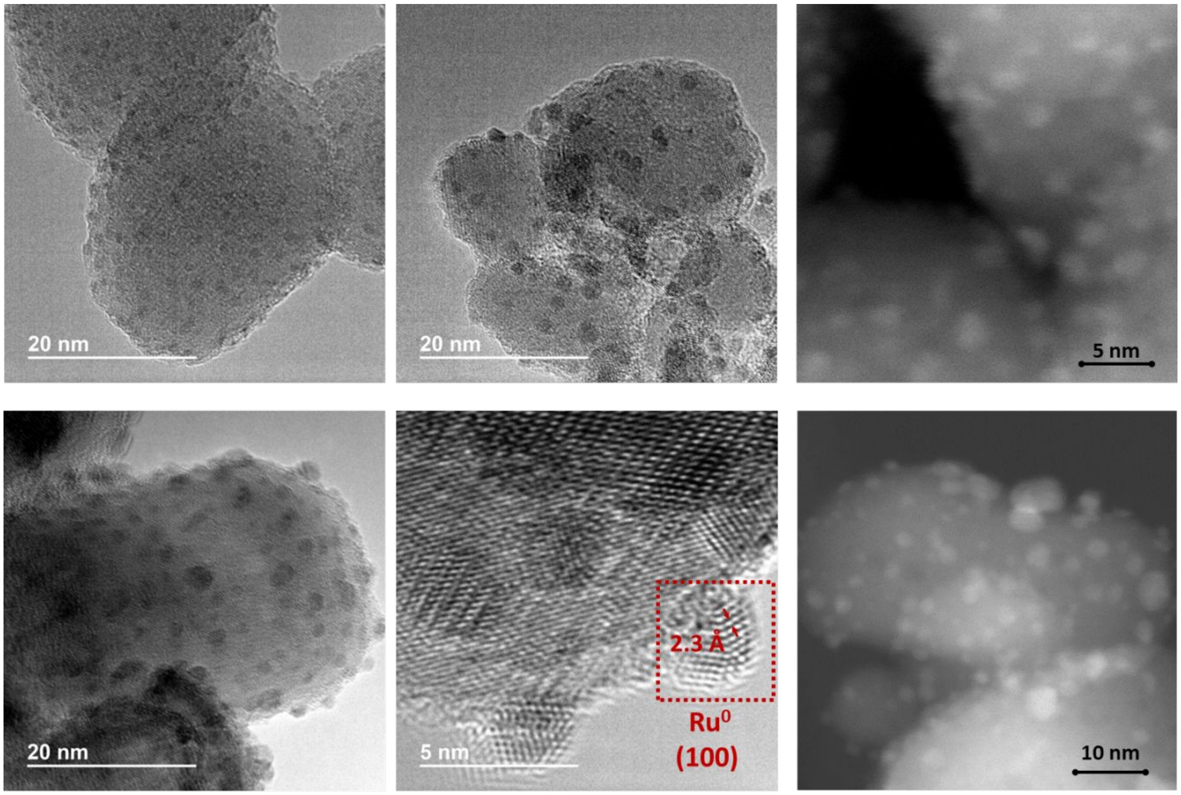

Figure S10. HRTEM and ADF-STEM images of the Ru/STO photocatalyst after 4 h (top images) and 96 h (bottom images) of continuous irradiation (9.86·W cm^{-2}) and gas feed.

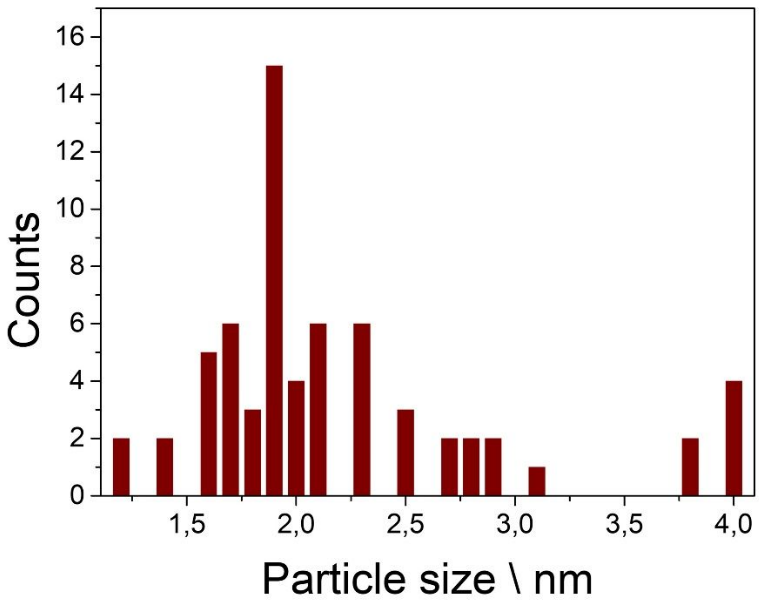

Figure S11. Particle size distribution of Ru/STO after 96 h of catalytic out under gas feed and 9.86 W·cm^{-2} light irradiation.

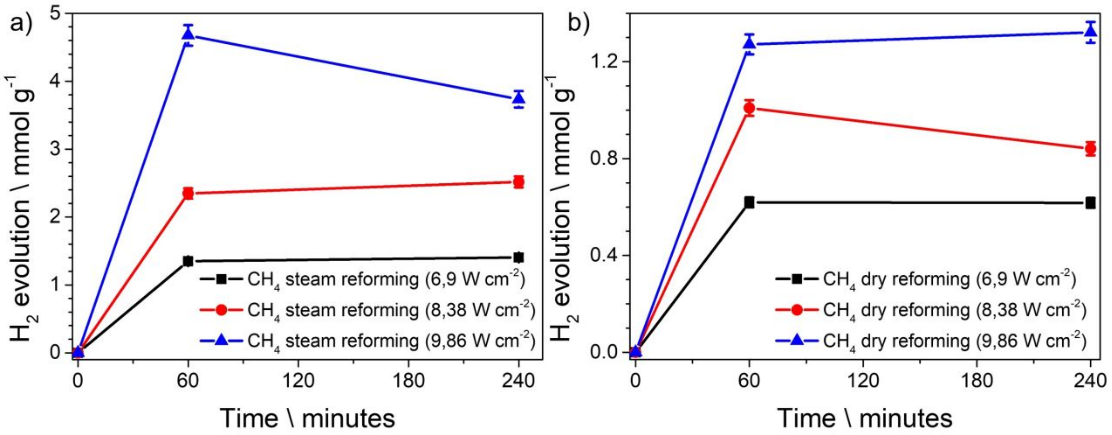

Figure S12. H_2 evolution from the steam (a) and dry (b) obtained from the reforming of CH_4 at different light intensities.

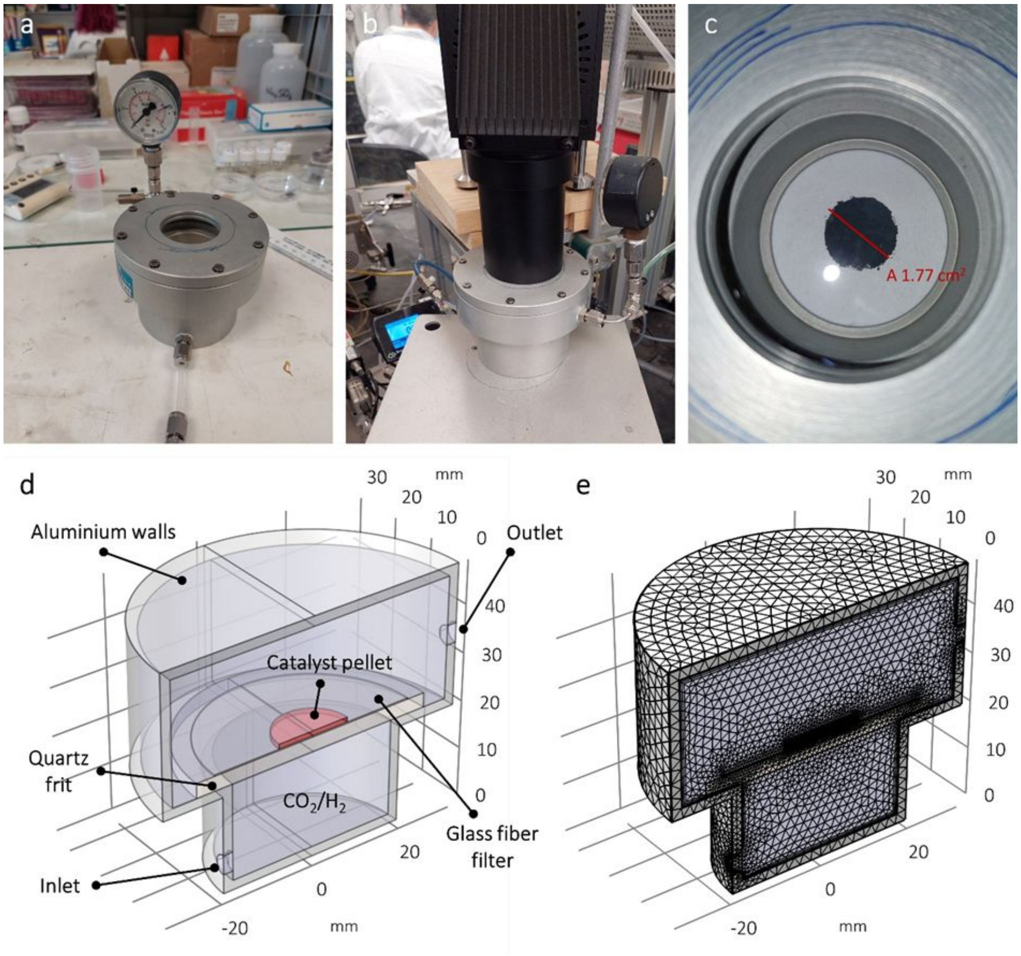

Figure S13. Photothermal reactor and corresponding COMSOL model. a) Photograph of the reactor. b) Photograph of the reactor and lamp. c) Photograph of the catalyst pellet inside the reactor, on top of the quartz frit and glass fiber filter. d) COMSOL model geometry. e) Finite element mesh used for solving the COMSOL model.

The custom-built aluminum photoreactor used for the photocatalytic experiments was replicated in COMSOL Multiphysics as depicted in Figure S6. To reduce computational time, only half of the reactor was simulated while applying symmetry boundary conditions to the internal plane of symmetry. The inlet and outlet gas tubes were modelled as 4 mm diameter round holes. Within the reactor, the catalyst was modelled as a disk (1 mm thickness, 15 mm diameter), placed on top of a circular glass fiber filter (0.26 mm thickness, 42.5 mm diameter), on top of a circular glass frit (3 mm thickness, 50 mm diameter).

For all used materials (aluminum, quartz glass, strontium titanate, hydrogen, and carbon dioxide) the temperature-dependent density, thermal conductivity, heat capacity, and dynamic viscosity from the

built-in material library were used. The effective material properties of the gas mixture (1:1 CO_2:H_2) were taken as the volumetric averages of the two gas constituent properties. The presence of 2.5 wt.% Ru was assumed to have a negligible influence on the thermal material properties of the $SrTiO_3$ catalyst solid.

Physics modelling

The heat transfer and fluid flow within the reactor was simulated using the non-isothermal flow multiphysics interface, which coupled the "heat transfer in solids and fluids" module to simulate convective and conductive heat transfer and a "laminar flow" module to simulate the fluid dynamics. The temperature of the external aluminum reactor wall was fixed according to *operando* thermal probe measurement at the highest illumination intensity (9.86 W·cm^{-2}), determined to be 40 °C. For lower illumination intensities the external wall temperature scaled linearly, according to the illumination intensity, between 20°C and 40°C.

The photothermal heating was included as a boundary heat source ("Deposited beam power") with a Gaussian distribution (0.9 mm standard deviation) and a deposited beam power equal to the product of the illumination intensity and the fraction of absorbed light (94%, as determined by diffuse reflectance measurements).

The gas mixture was flowed into the reactor as a "fully developed flow" through an inlet on the left bottom side at a flow rate of 26 mL·min^{-1} (13 mL·min^{-1} for the model cut in half), and flowed out of the reactor as a "fully developed flow" through an outlet at the top right, where the boundary condition was set to an average pressure of 0 Pa. A "no slip" boundary condition was applied to all interior aluminum walls.

Fluid dynamics through the porous catalyst pellet, filter, and frit were modelled with the Brinkman equations by adding a "Fluid and Matrix Properties" node to the laminar flow module. The physical model was set to a compressible flow (Ma<0.3), gravity was included, and the reactor was kept under atmospheric pressure. The gas permeability of the quartz frit (k, in m^2) was estimated from the porosity (porous volume fraction, ε = 0.63) [8], the average pore size (D = 160 – 250 µm)[9], and the experimentally determined tortuosity (T = 1.4)[8] according to a variant of the Kozeny-Carman equation[10,11]:

$$k = \frac{\varepsilon D^2}{32T}$$

For the quartz frit, this amounted to k = 5.6 × 10^{-10} m^2. Similarly, the gas permeability of the glass microfiber filter (Whatman art. no. 1820-042) was calculated from the estimated porosity (based on dimensions and weight, ε = 0.91), specified pore volume (D = 1.6 μm), and an estimated tortuosity (T = 4) to be 2 × 10^{-14} m^2. Finally, the catalyst pellet had an estimated porosity of 0.88 (based on pellet dimensions and weight, and the density of strontium titanate) and, because the pellet was not compressed, an estimated permeability of 1 × 10^{-12} m^2.

As described by Un and coworkers[12], the thermal conductivity of catalyst nanopowders does not scale as the volume-weighted average of the two components because the pellet consists of many loosely connected nanoparticles. Therefore, the effective thermal conductivity is dominated by that of the gas phase[12]. To this end, the effective thermal conductivity of the pellet was approximated by the weighted geometric mean of gas and porous matrix conductivities (the "Power law" option in COMSOL):

$$\kappa_{eff} = {\kappa_{STO}}^{\theta_s} \cdot {\kappa_{gas}}^{1-\theta_s}$$

where κ_{STO} is the built-in thermal conductivity of $SrTiO_3$, κ_{gas} is the effective thermal conductivity of the CO_2/H_2 mixture, and θ_s is the solid volume fraction (that is, $\theta_s = 1 - \varepsilon$).

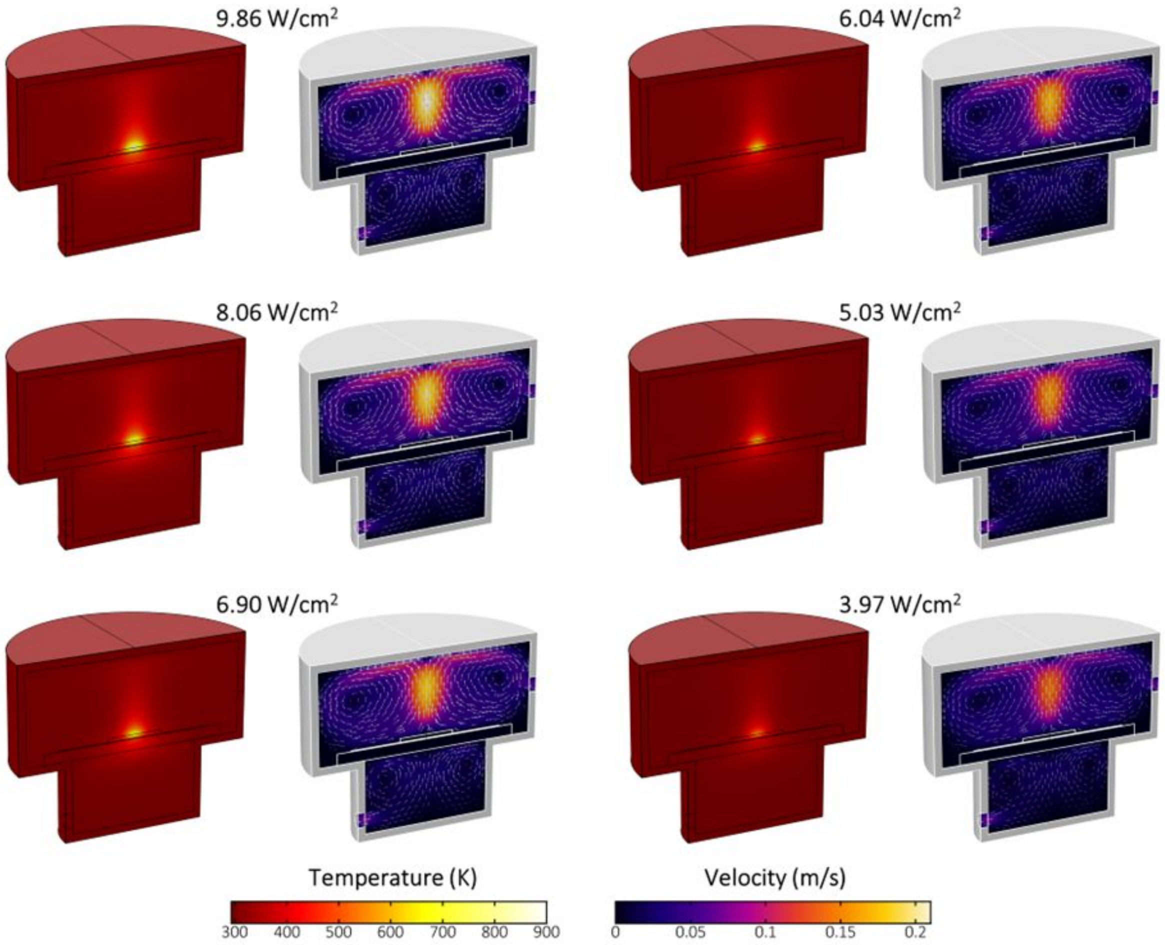

Figure S14. Temperature distributions and gas velocity inside the photothermal reactor at the six different experimental illumination intensities.

Diffuse reflectance measurements

Diffuse reflectance (DR) spectra were collected on an Agilent Cary 5000 UV-Vis spectrometer (version 3.07) fitted with an integrating sphere. The catalyst powder was placed inside a custom-built powder cell, in which the powder sample was placed in a rubber O-ring that was sandwiched between two quartz windows, and clamped in a 3D-printed port cover and sample holder, which was painted with barium sulfate paint (Avian B coating)[13], see Figure S10. As near 100% DR reference sample, a white plastic disk was used that was painted with Avian B coating, which has a known diffuse reflectance spectrum[13]. As a near-0% DR reference sample, a black-painted plastic foam was used. These reference samples were measured in the same custom-built powder cell as the catalyst samples, with omission of the rubber O-ring. The small spot accessory kit was used to focus the light beam well within the sample surface area.

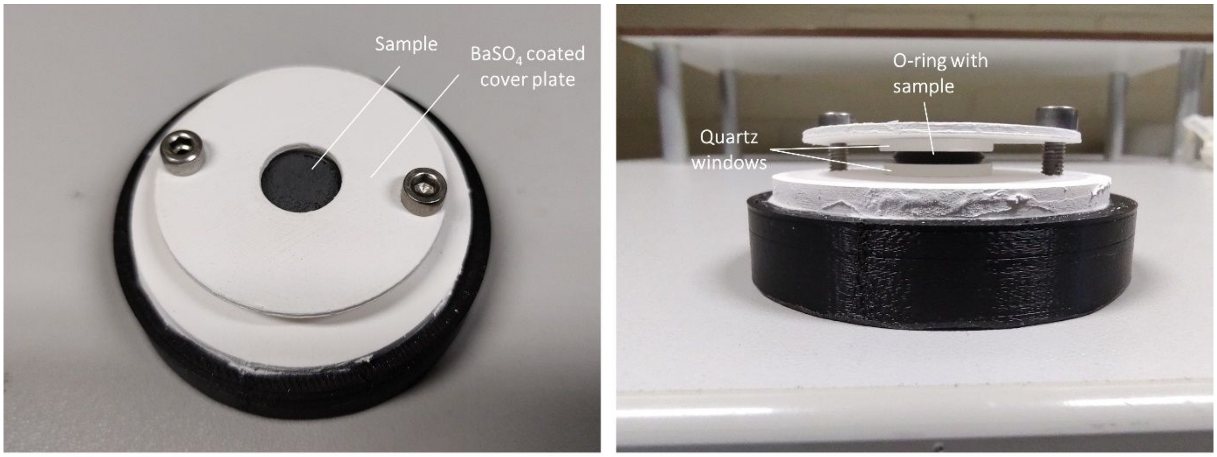

Figure S15. Geometry of the custom-built powder-cell for diffuse reflectance measurements.

DR spectra were collected from 350 – 870 nm with a 1 nm data interval and a minimum signal-to-noise ratio of 200 per collected point. The spectrometer operated in dual-beam mode, where the spectra were referenced against a reference beam that hit a 100% diffuse reflectance standard on a separate port in the integrating sphere. Finally, the collected sample spectra were corrected for light losses and specular reflections of the powder cell by the following formula:

$$DR_{sample,\, corrected}(\lambda) = \left(DR_{sample}(\lambda) - DR_{black}(\lambda)\right) * \frac{DR_{ref}(\lambda)}{DR_{white}(\lambda) - DR_{black}(\lambda)}$$

Here, subscripts “black” and “white” denote the near-0% and near-100% DR references, and subscript “ref” refers to the reference spectrum supplied by the manufacturer. The corrected DR spectra, shown in Figure S5, indicate that unfunctionalized STO reflects >90% of the incident light beyond the UV bandgap, while the Ru-STO pellet only reflects 5 – 10%, that is, it absorbs 90 – 95% of the incident light.

Finally the fraction of absorbed simulated sunlight (F_{abs}) was calculated by integrating the product of the spectral absorptance and the AM 1.5G irradiance spectrum of the sun (I_{sun} , direct + circumsolar, in $W/(m^2.nm)$) from NREL[14]:

$$F_{abs} = \frac{\int I_{sun}(\lambda) * (1 - DR_{sample,corrected}(\lambda)\,)}{\int I_{sun}(\lambda)}$$

From this calculation it was determined that Ru-STO absorbed 94% of the incident simulated sunlight, which was used in the COMSOL model to simulate the local temperature distribution within the catalyst pellet.

References

(1) Peng, Y.; Albero, J.; Franconetti, A.; Concepción, P.; García, H. Visible and NIR Light Assistance of the N2 Reduction to NH3 Catalyzed by Cs-Promoted Ru Nanoparticles Supported on Strontium Titanate. *ACS Catal* **2022**, *12* (9), 4938–4946. https://doi.org/10.1021/acscatal.2c00509.

(2) Mateo, D.; Albero, J.; García, H. Titanium-Perovskite-Supported RuO2 Nanoparticles for Photocatalytic CO2 Methanation. *Joule* **2019**, *3* (8), 1949–1962. https://doi.org/https://doi.org/10.1016/j.joule.2019.06.001.

(3) Wu, Z.; Shen, J.; Li, C.; Zhang, C.; Feng, K.; Wang, Z.; Wang, X.; Meira, D. M.; Cai, M.; Zhang, D.; Wang, S.; Chu, M.; Chen, J.; Xi, Y.; Zhang, L.; Sham, T.-K.; Genest, A.; Rupprechter, G.; Zhang, X.; He, L. Mo2TiC2 MXene-Supported Ru Clusters for Efficient Photothermal Reverse Water–Gas Shift. *ACS Nano* **2023**, *17* (2), 1550–1559. https://doi.org/10.1021/acsnano.2c10707.

(4) Dong, T.; Liu, X.; Tang, Z.; Yuan, H.; Jiang, D.; Wang, Y.; Liu, Z.; Zhang, X.; Huang, S.; Liu, H.; Zhao, L.; Zhou, W. Ru Decorated TiOx Nanoparticles via Laser Bombardment for Photothermal Co-Catalytic CO2 Hydrogenation to Methane with High Selectivity. *Appl Catal B* **2023**, *326*, 122176. https://doi.org/https://doi.org/10.1016/j.apcatb.2022.122176.

(5) Kong, N.; Han, B.; Li, Z.; Fang, Y.; Feng, K.; Wu, Z.; Wang, S.; Xu, A.-B.; Yu, Y.; Li, C.; Lin, Z.; He, L. Ruthenium Nanoparticles Supported on Mg(OH)2 Microflowers as Catalysts for Photothermal Carbon Dioxide Hydrogenation. *ACS Appl Nano Mater* **2020**, *3* (3), 3028–3033. https://doi.org/10.1021/acsanm.0c00383.

(6) Kim, C.; Hyeon, S.; Lee, J.; Kim, W. D.; Lee, D. C.; Kim, J.; Lee, H. Energy-Efficient CO2 Hydrogenation with Fast Response Using Photoexcitation of CO2 Adsorbed on Metal Catalysts. *Nat Commun* **2018**, *9* (1), 3027. https://doi.org/10.1038/s41467-018-05542-5.

(7) Liu, X.; Xing, C.; Yang, F.; Liu, Z.; Wang, Y.; Dong, T.; Zhao, L.; Liu, H.; Zhou, W. Strong Interaction over Ru/Defects-Rich Aluminium Oxide Boosts Photothermal CO2 Methanation via Microchannel Flow-Type System. *Adv Energy Mater* **2022**, *12* (31), 2201009. https://doi.org/https://doi.org/10.1002/aenm.202201009.

(8) Kim, Y.; Gostick, J. T. Measuring Effective Diffusivity in Porous Media with a Gasket-Free, Radial Arrangement. *Int J Heat Mass Transf* **2019**, *129*, 1023–1030. https://doi.org/https://doi.org/10.1016/j.ijheatmasstransfer.2018.10.054.

(9) Technical Glass Products. *Glass Frits*. https://technicalglass.com/product-pages/quartz-frits/ (accessed 2023-07-27).

(10) Maurath, J.; Dittmann, J.; Schultz, N.; Willenbacher, N. Fabrication of Highly Porous Glass Filters Using Capillary Suspension Processing. *Sep Purif Technol* **2015**, *149*, 470–478. https://doi.org/https://doi.org/10.1016/j.seppur.2015.06.022.

(11) F. A.L. Dullien. *Porous Media: Fluid Transport and Pore Structure*; Academic Press: New York, 1979.

(12) Un, I. W.; Dubi, Y.; Sivan, Y. Photothermal Nonlinearity in Plasmon-Assisted Photocatalysis. *Nanoscale* **2022**, *14* (13), 5022–5032. https://doi.org/10.1039/D1NR07822D.

(13) Avian-B White Reflectance Coating. *Avian Technologies.https://aviantechnologies.com/product/avian-b-white-reflectance-coating/ (accessed 2023-07-28).*

(14) Reference Air Mass 1.5 Spectra. *https://www.nrel.gov/grid/solar-resource/spectra-am1.5.html (accessed 2023-07-28).*
